# Fast expansion and slow contraction of the ITCZ in response to CO_2_ forcing

**DOI:** 10.1126/sciadv.aef2833

**Published:** 2026-07-23

**Authors:** Jiayu Zhang, Sarah M. Kang

**Affiliations:** Max Plank Institute for Meteorology, Hamburg, Germany.

## Abstract

The Intertropical Convergence Zone (ITCZ) strongly shapes tropical rainfall and global circulation, yet its response to carbon dioxide (CO_2_) forcing remains uncertain. Climate models project a long-term narrowing of the ITCZ under warming, but recent decades instead show a poleward expansion linked to eastern tropical Pacific cooling. Using a 30-member ensemble of abruptly quadrupled CO_2_ experiments, we identify a two-phase evolution: a fast expansion over the first decade, driven by basin-wide relative cooling in the equatorial Pacific, followed by a slow contraction as equatorial warming strengthens. Applying this framework to a 1%-per-year CO_2_ scenario reveals that the slow phase dominates only after ∼80 years, when the CO_2_ levels more than double relative to preindustrial, suggesting that present-day conditions are still dominated by the fast response. This potentially reconciles recent ITCZ expansion with long-term projections of contraction, underscoring the importance of accounting for timescale-dependent processes when interpreting past trends and projecting near-term climate change.

## Teaser

The ITCZ expands fast and contracts slow under CO_2_ forcing, driven by equatorial Pacific SST warming shift.

## INTRODUCTION

The Intertropical Convergence Zone (ITCZ), a narrow belt of intense updraft and precipitation encircling the equator, exerts a fundamental control on the tropical rainfall and global circulation. Deep convection at its core drives teleconnections via Rossby wave trains that propagate into the midlatitudes of both hemispheres, shaping many aspects of global climate (*1*, *2*). In particular, variations in ITCZ width affect the extent of the tropics, the position of storm tracks (*1*), the distribution of global precipitation (*3*), and climate sensitivity (*4*, *5*). Its width thus represents a key controlling feature of Earth’s climate system, and understanding how it responds to CO_2_ forcing is critical for anticipating future regional and global climate impacts.

**Fig. 1.**
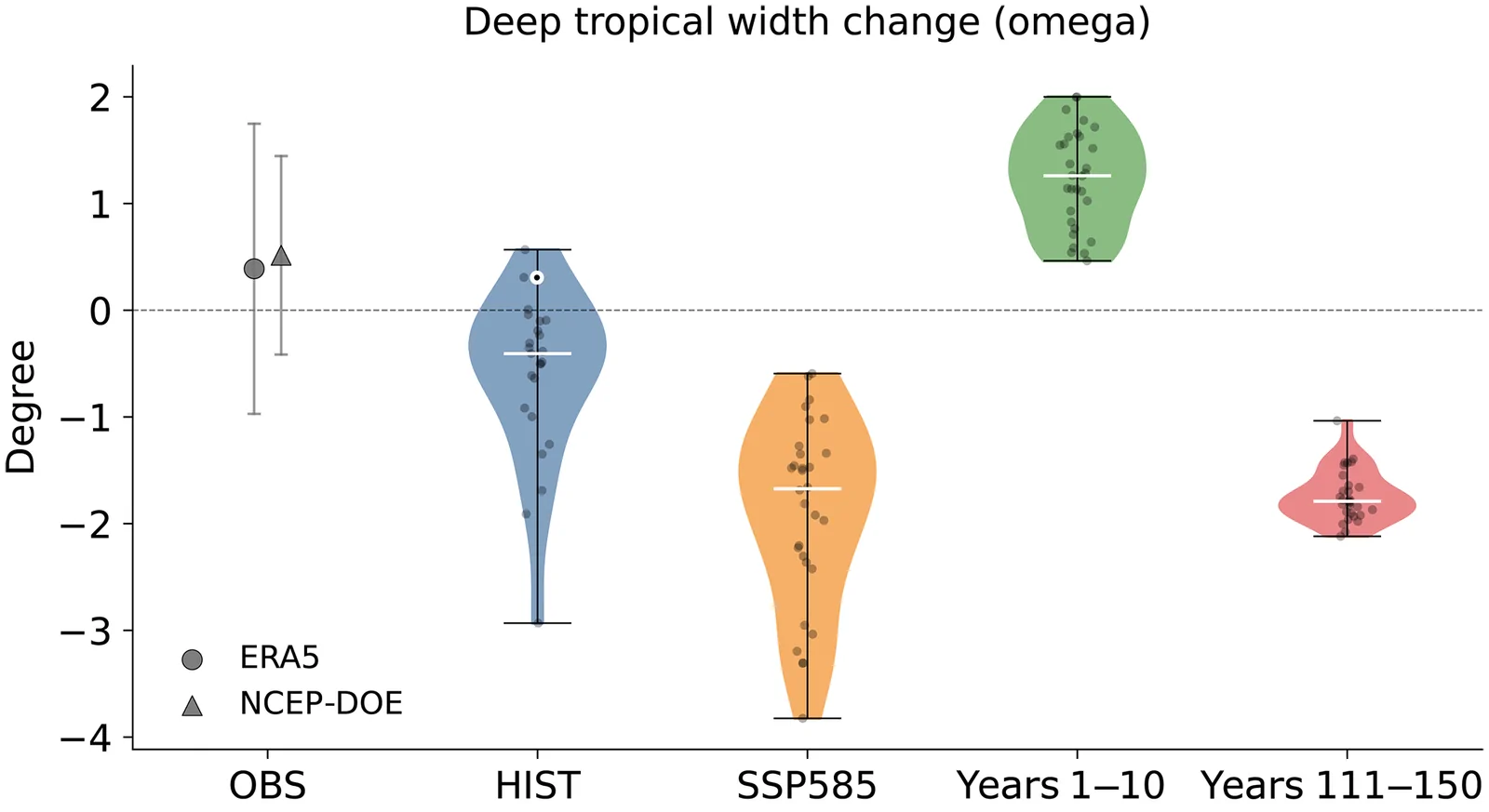
Deep-tropical width changes in different scenarios. The deep-tropical width ($\phi_{\text{width}}$) is defined as the distance between the centroids of the zonal-mean pressure velocity (averaged between 300 and 700 hPa) in the Northern and Southern hemispheres (Materials and Methods and fig. S3). The difference in deep tropical width is shown for the reanalysis data [ERA5 (*39*) and NCEP-DOE (*40*)] between the most recent decade (2015–2024) and the earlier decade (1979–1988) (observations, OBS); for the CMIP6 models over the same periods, derived from the combined historical and SSP5-8.5 scenarios (HIST); and for the future projection (SSP585), calculated as the difference between the years 2080–2099 and the last 200 years of the preindustrial state. The error bars in the first column show the 90% confidence intervals for the difference between the two decades (∆), estimated as $∆\pm t_{\alpha /2}\sqrt{\frac{S_{x}^{2}}{N_{x}}+\frac{S_{y}^{2}}{N_{y}}}$, where $N_{x}$ and $N_{y}$ are the number of years in each decade, $S_{x}$ and $S_{y}$ are the sample variances, and $t_{\alpha /2}$ is the critical value of the Student’s *t* distribution at a significance level of α=0.1. Dots in the second and third columns indicate individual models. The last two columns denote the first 10-year (years 1–10) and last 40-year (years 111–150) anomalies relative to the preindustrial state in our 30-member abrupt4xCO_2_ experiments. The white horizontal line in each column denotes the ensemble median, and the shading denotes the probability density distribution. In HIST, the MPI-ESM-LR result is marked by a white contour. Positive and negative values represent expansion and contraction of the deep tropical width, respectively.

It is well established that the ITCZ narrows under global warming (*6*), with this “deep tropics squeeze” emerging as a robust projection across climate models (yellow column in Fig. 1 and fig. S1) While dynamical constraints on the ITCZ width exist (*7*–*10*), its narrowing in response to increased CO_2_ is often attributed to the amplified equatorial surface warming (fig. S2A) (*11*, *12*). In contrast to future projections, observations do not show a large narrowing signal, despite modest global warming in recent decades, regardless whether the deep tropical width is defined using circulation-based (*6*, *7*, *11*, *13*) or precipitation-based (*14*–*16*) metrics (first column in Fig. 1 and fig. S1). A slight poleward expansion in observations has been linked to cooling in the eastern equatorial Pacific (fig. S2B) (*17*). This highlights the critical role of the tropical sea surface temperature (SST) pattern in controlling the ITCZ width. Since the tropical SST pattern itself evolves with increasing CO_2_, from an initial phase of relative cooling in the eastern Pacific, often attributed to the ocean dynamical thermostat (*18*, *19*), to a later phase of amplified equatorial warming (*20*), the ITCZ may first expand before transitioning to the well-known squeeze. Such a potential expansion in the fast response to CO_2_ could partially account for the recently observed ITCZ widening.

Together, the previously reported robust “deep tropics squeeze” may reflect the focus of most studies on long-term changes over multidecadal to centennial timescales. In contrast, the fast response to CO_2_ forcing may involve an expansion of the ITCZ. The climate system is known to exhibit distinct responses at different timescales, as shown for the midlatitude jet and Hadley cell edge, surface warming patterns, and Mediterranean precipitation (*21*–*24*). Extending this perspective to the ITCZ suggests that its width may evolve in qualitatively different ways from the fast to the long-term response. Understanding this evolution is crucial for improving confidence in near-term tropical climate projections.

Here, we investigate the evolution of ITCZ width using a large ensemble of abruptly quadrupled CO_2_ (abrupt4xCO_2_) experiments, which enables a robust quantification of the timescales of forced response. We find that the ITCZ initially expands in response to CO_2_ forcing, driven by relative cooling in the eastern tropical Pacific, before contracting in the long term as the tropical SST pattern shifts toward amplified equatorial warming. The transition in relative SST changes in the equatorial Pacific, from cooling to warming, is a key driver of ITCZ width evolution through its influence on the atmospheric energy budget (*11*, *25*).This two-phase evolution highlights the need to account for timescale-dependent processes when interpreting ITCZ changes in both historical records and future projections.

## RESULTS

### Contrasting CO_2_-driven fast and long-term responses in the deep tropics

**Fig. 2.**
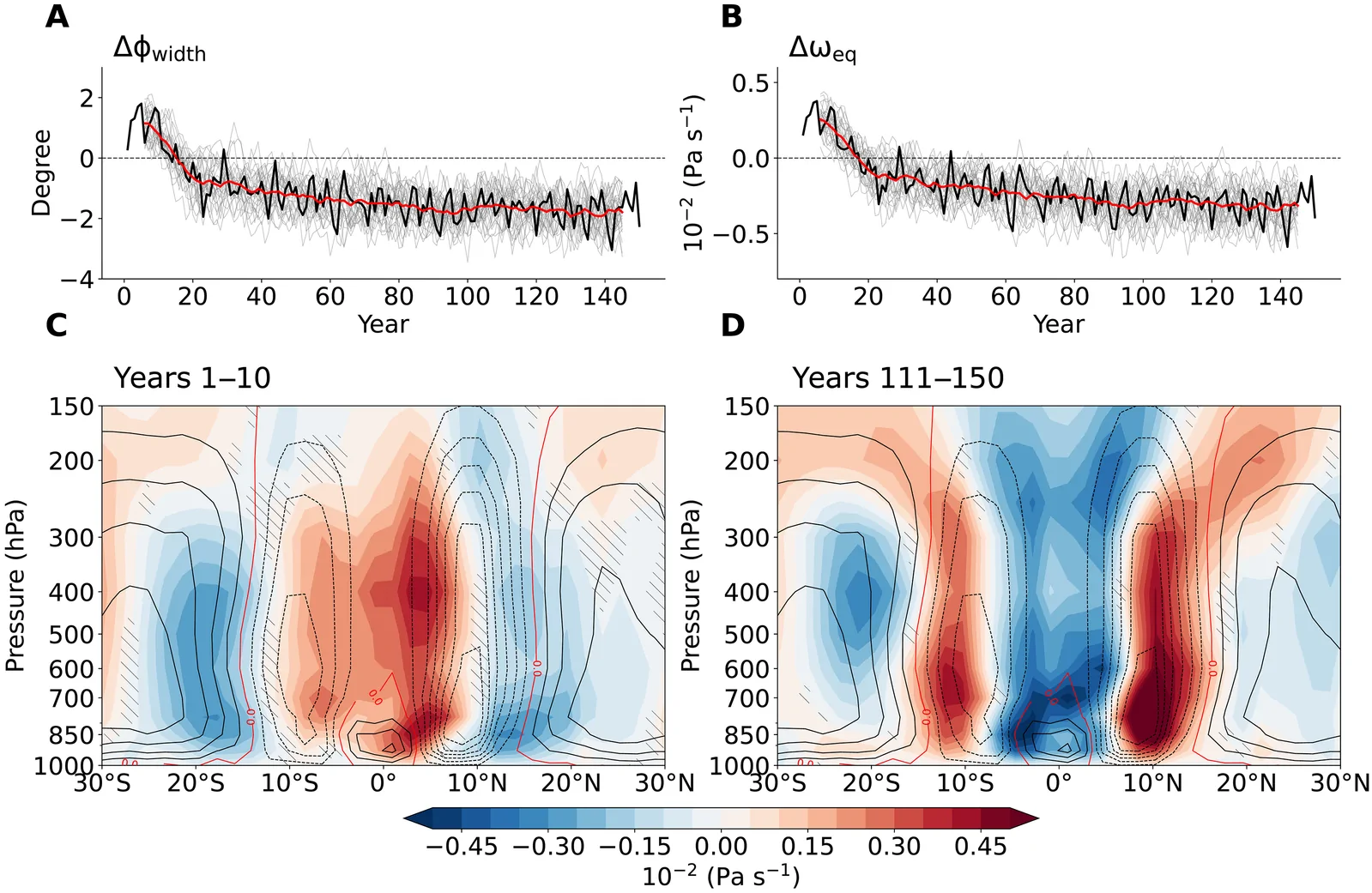
Evolution of tropical vertical motion in the abrupt4xCO_2_ simulation. Time series of (**A**) deep tropical width ($\phi_{\text{width}}$; positive values indicate poleward expansion and negative values contraction) and (**B**) equatorial (5°S to 5°N) pressure velocity anomalies averaged between 300 and 700 hPa ($\omega_{\text{eq}}$; positive values indicate weakening and negative values strengthening). Gray lines show the 11-year running mean of individual ensemble members, black the annual 30-member mean, and red its 11-year running mean. Pressure velocity anomalies for the (**C**) fast (years 1–10) and (**D**) long-term (years 111–150) responses. Regions with values not significant at the 95% confidence level are hatched according to the *t* test. Contours show the preindustrial climatology (ascent in dashed; descent in solid).

We construct a 30-member abrupt4xCO_2_ experiments with Max Planck Institute Earth System Model, Low Resolution (MPI-ESM-LR) (*26*) to isolate the forced response. Our focus is on the width of the deep tropics (*14*), defined as the distance between the centroids of the zonal-mean pressure velocity (averaged between 300 and 700 hPa) in the two hemispheres, denoted as $\phi_{\text{width}}$ (Materials and Methods and fig. S3). We examine the response to abrupt CO_2_ quadrupling relative to the preindustrial climatology. The time series of changes in $\phi_{\text{width}}$ reveal a two-phase evolution: a fast expansion followed by a slow contraction (Fig. 2A). This transition is also evident when defining the ITCZ width as the distance between the northern and southern edges of upward motion (fig. S4) (*7*). We define the fast response as the mean over years 1–10 and the long-term response as the mean over years 111–150, when the response is quasi-equilibrated. In the fast response, changes in the latitude-height structure of pressure velocity indicate a widening of the deep tropics accompanied by weakened equatorial updraft (Fig. 2C), whereas in the long-term response, the deep tropics narrows and equatorial ascent strengthens (Fig. 2D). Consistently, the time series of equatorial updraft amplitude also exhibits a clear two-phase evolution, transitioning from weakening to strengthening (Fig. 2B). The fast expansion and subsequent long-term contraction are robust across all ensemble members (green and red columns in Fig. 1), and similar results are obtained when the deep tropical width is defined using precipitation (figs. S1 and S5). While the timescale of the fast response of the ITCZ width is on the order of 10 years, that of the ITCZ location is suggested to operate on a centennial timescale (*27*). The large 30-member ensemble is crucial to robustly separate the forced signal from internal variability. The minimum ensemble size required to detect the forced changes in both deep tropical width and equatorial vertical motion over 150 years is greater than 10 but smaller than 30 (fig. S6), demonstrating that an ensemble of 30 is not only sufficient but also necessary to ensure robustness.

**Fig. 3.**
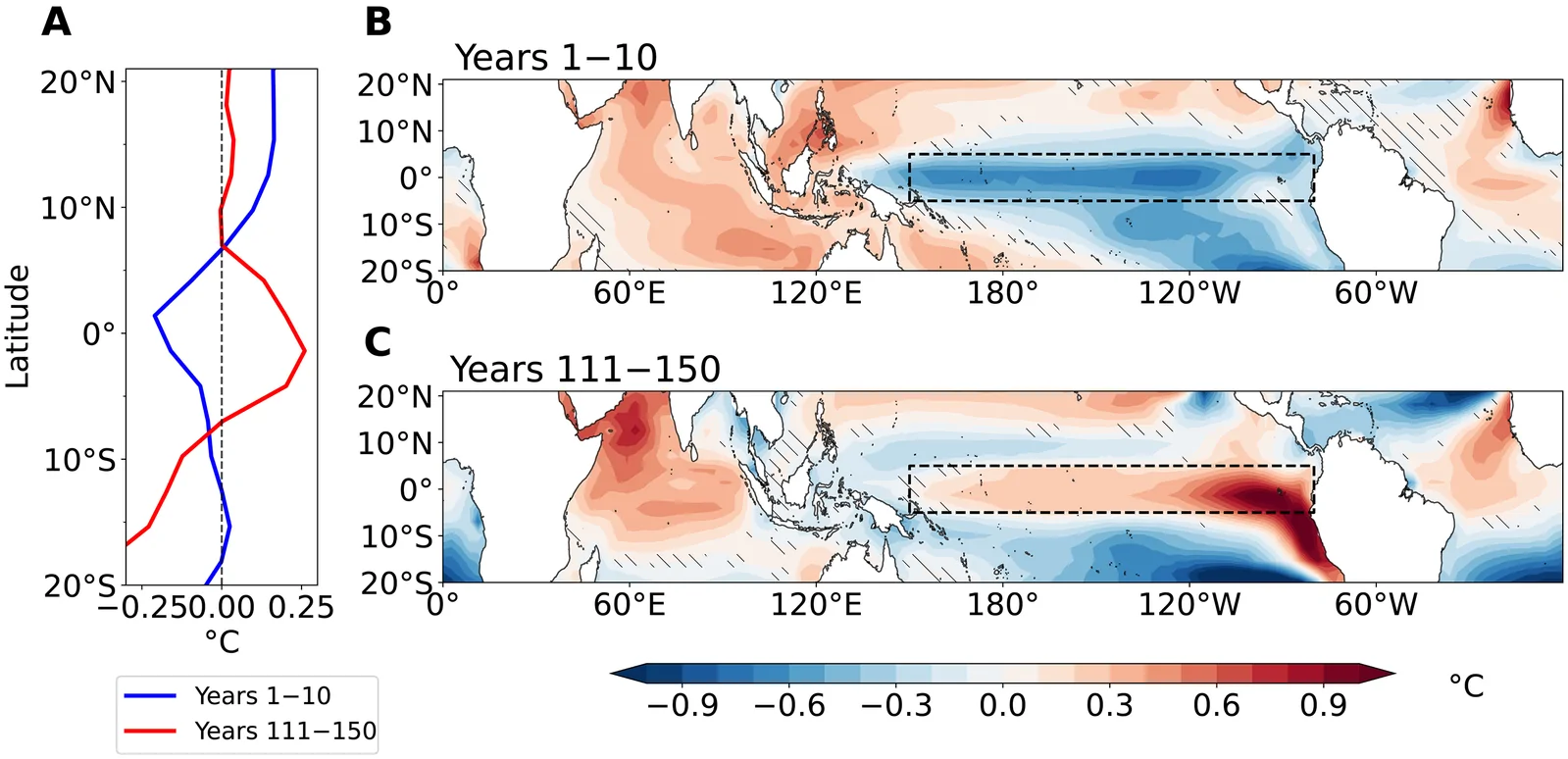
Fast and long-term responses of relative tropical SST. (**A**) Zonal-mean SST changes, after removing the average over 20°S to 20°N, in the fast (years 1–10; blue) and long-term (years 111–150; red) periods. Spatial patterns of relative SST anomalies for the (**B**) fast and (**C**) long-term responses. Hatched regions indicate where values are not significant at the 95% confidence level according to the *t* test. The dashed box marks the region used to calculate the regional mean in Fig. 4.

We now move to examine the evolution of tropical SST warming pattern, a key factor controlling changes in both the intensity of vertical motion and the width of the deep tropics (*11*, *28*). In the fast response, the deep tropics (5°S to 5°N) exhibit cooling relative to the tropical mean, which transitions to relative warming in the quasi-equilibrium state (Fig. 3A), consistent with the shift in equatorial ascent from weakening to strengthening (Fig. 2B). Spatially, these zonal-mean SST changes are dominated by responses over the equatorial Pacific, with relative cooling across the basin in the fast response (Fig. 3B) and enhanced warming, peaking in the eastern basin, in the long-term response (Fig. 3C).

Relative SST changes in the equatorial Pacific have been identified as a key driver of ITCZ width changes through their influence on the atmospheric energy budget (*11*, *17*). Enhanced equatorial Pacific warming is associated with increased net energy input into the atmospheric column and reduced gross moist stability, as the resulting increase in lower-tropospheric moist static energy exceeds that in the upper troposphere (fig. S7, A, C, D, and F). This energetically leads to an equatorward shift of peak ascent (*11*, *17*, *29*). While this mechanism is well established, we show that, in the fast response, relative cooling in the equatorial Pacific exerts the opposite effect (fig. S7, A, B, D, and E). The strong control of the tropical Pacific SST warming pattern on the deep tropical width and equatorial vertical motion is further corroborated by the intermodel relationship across Coupled Model Intercomparison Project phase 6 (CMIP6), which exhibits correlations as high as 0.9 (fig. S8).

**Fig. 4.**
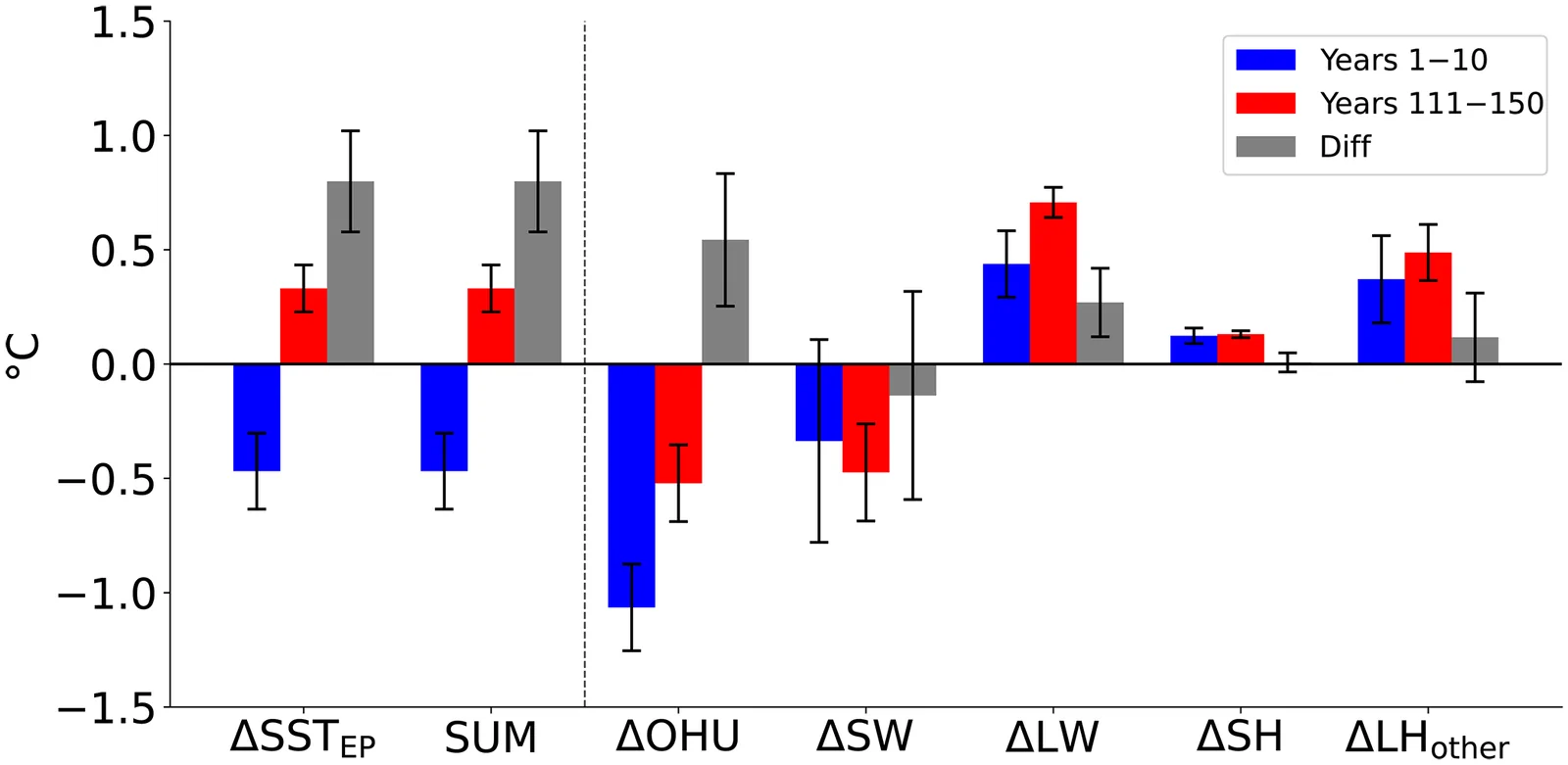
Ocean mixed layer heat budget for relative SST response in equatorial Pacific. Relative SST response averaged over 5°S to 5°N and 150°E to 80°W and its decomposition into contributions from changes in ocean heat uptake (OHU), shortwave radiation (SW), longwave radiation (LW), sensible heat flux (SH), and latent heat flux related to wind and relative humidity (LH_other_). The close match between the sum of all components (SUM) and the actual response ($∆{\text{SST}}_{\text{EP}}$) confirms the validity of the decomposition. Blue bars indicate the fast response (years 1–10), red bars the long-term response (years 111–150), and gray bars the difference (Diff) between the two. Error bars denote 1.5 times the standard deviation of 30 ensembles.

Changes in the tropical SST warming pattern can be understood from the ocean mixed-layer heat budget (Materials and Methods, Fig. 4, and fig. S10). The relative equatorial Pacific cooling in the fast response (blue in Fig. 4) is primarily driven by the change in ocean heat uptake. In contrast, the relative warming in the long-term response (red in Fig. 4) arises from weakened evaporation due to reduced trade easterlies, with this effect further amplified by longwave feedback. The difference between the long-term and fast responses (gray in Fig. 4) is dominated by evolving changes in ocean heat uptake, whose spatial pattern exhibits basin-wide warming in the equatorial Pacific (fig. S10). Hence, the evolution of ocean dynamical adjustments is the primary driver of the transition in the tropical Pacific warming pattern, from fast-phase cooling to long-term warming, and, in turn, a key factor controlling the shift in deep tropical width.

### Transient evolution in the 1%CO_2_ simulation

One might wonder how the two-time scale evolution in the idealized abrupt4xCO_2_ simulation relates to a more realistic forcing scenario. To address this, we examine the 1%-per-year CO_2_ increase simulation. The climate response in this scenario can be interpreted as a linear combination of the fast and slow components derived from the abrupt4xCO_2_ experiment, where the slow component is defined as the difference between the long-term and fast responses. By regressing the 1%-per-year CO_2_ response onto the fast and slow patterns identified from abrupt4xCO_2_, we decompose the total response into contributions from each component (Materials and Methods).

**Fig. 5.**
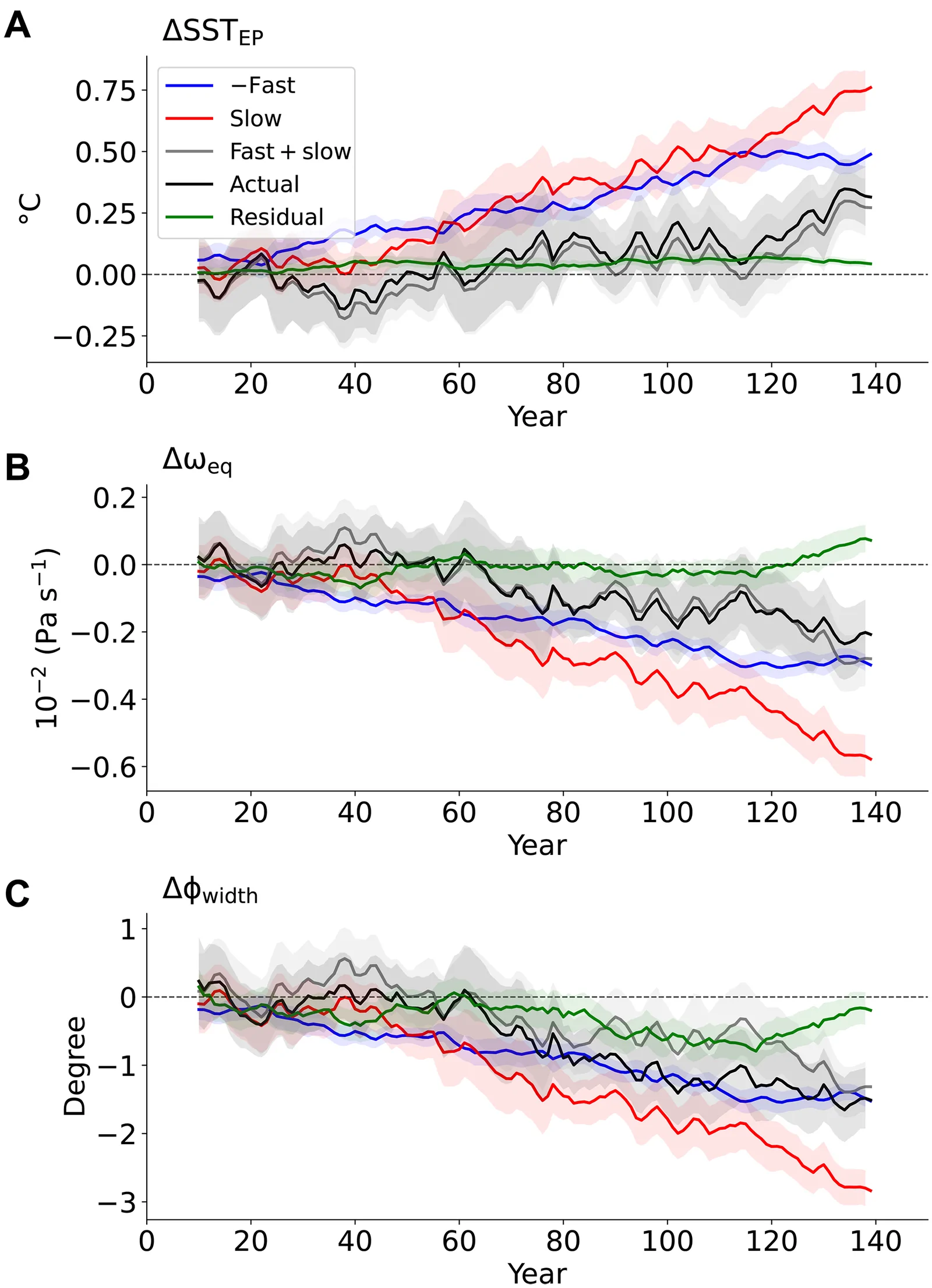
Time series of responses in 1%-per-year CO_2_ simulation. Anomalies in (**A**) relative SST in the equatorial Pacific $∆{\text{SST}}_{\text{EP}}$, (**B**) equatorial (5°S to 5°N) pressure velocity averaged between 300 and 700 hPa $∆\omega_{\text{eq}}$, and (**C**) deep tropical width $∆\phi_{\text{width}}$. The blue represents the fast component with reversed sign (for direct comparison with the slow component), red the slow component, gray the sum, black the actual anomalies, and green the residual. All lines are filtered with a 21-year running mean. Shading indicates the 95% confidence interval based on variability within each 21-year window.

We can expect the fast component to dominate in the early period of the simulation, with the fractional contribution of the slow component gradually increasing over time. Indeed, the shift from the dominance of the fast response (blue in Fig. 5) to dominance of the slow response (red in Fig. 5) is evident in the relative SST anomaly in the equatorial Pacific, the amplitude of equatorial ascent, and the deep tropical width. The residual of the deep tropical width change is larger than the other two variables (green in Fig. 5). This likely reflects the additional reconstruction steps required to diagnose ITCZ width (Materials and Methods). As discussed earlier, the fast response is characterized by relative cooling in the equatorial Pacific, weakened equatorial ascent, and an expansion of the deep tropics, whereas the slow response features relative warming in the equatorial Pacific, intensified equatorial ascent, and a contraction of the deep tropics (fig. S11). In the 1%-per-year CO_2_ scenario, the early period shows insignificant net response because the fast and slow components largely cancel each other. The transition occurs around year 60, and it is only after around year 80, when the slow component becomes substantially larger than the fast component, that the CO_2_-forced slow response in the 1%-per-year CO_2_ simulation becomes evident (fig. S12).

The timing at which the slow component becomes detectable (around year 80) corresponds to a CO_2_ increase by a factor of 2.2 relative to the preindustrial level. Given that the current CO_2_ level is ∼1.4 times the preindustrial level, present-day conditions are likely still dominated by the fast response. This may explain why observations over recent decades show cooling in the eastern tropical Pacific and an expansion of the deep tropics, features that differ from long-term model projections. If CO_2_ concentrations continue to rise at 1% per year, the tropical SST warming pattern is expected to remain in a La Niña–like state, accompanied by continued expansion of the deep tropics for roughly the next 50 years. However, this estimate is based solely on the effect of increasing CO_2_. In reality, the evolution of aerosol forcing (*30*), the influence of Antarctic meltwater (*31*), stratospheric ozone (*32*), and internal variability (*33*) also play important roles, adding substantial uncertainty to the timing of this transition.

## DISCUSSION

Using a large ensemble of abrupt4xCO_2_ simulations, we show that the deep tropical circulation undergoes a distinct two-phase evolution over the 150-year integration. In the first decade, the deep tropics experience weakened ascent and a widening of the deep tropics, followed by strengthened ascent and a narrowing of the deep tropics in subsequent decades. This reversal is linked to a shift in the SST warming pattern, from relative cooling to amplified warming in the equatorial region. Similar transitions in tropical SST patterns have been reported in a subset of CMIP models, which also exhibit equatorial Pacific cooling and an increased zonal SST gradient in the fast response, followed by a reversal in the long-term response (fig. S8) (*34*, *35*). The absence of this transition in other models may reflect the use of a single ensemble member (*19*) as internal variability may obscure the forced signal. The MPI-ESM-LR used in this study indicates that at least 10 members are needed to robustly detect the forced fast response (fig. S6). In addition, defining the fast response as the average over a fixed window (e.g., years 1–25) (*19*), as done in previous studies, may have masked transitions occurring at earlier stages. For example, in several CMIP6 models (BCC-CSM2-MR, FGOALS-f3-L, GISS-E2-1-H, and MRI-ESM2-0), shortening the time window from years 1–10 to years 1–3 reverses the tropical SST warming pattern (from slight enhanced warming to relative cooling) and the corresponding deep tropical width response (from weak equatorward contraction to poleward expansion) (fig. S9). Another possibility is that the fast response is inherently more variable across models than the long-term response. Together, these results highlight the need for a coordinated multimodel intercomparison of multimember abrupt4xCO_2_ experiments to robustly assess the transitional behavior.

The ocean dynamical thermostat is often invoked to explain the temporal evolution of CO_2_-induced warming patterns, whereby climatological upwelling of cold water over enhanced stratification, caused by delayed subsurface relative to surface warming, initially cools the equatorial Pacific (*18*). However, in our simulations, changes in ocean heat uptake produce basin-wide cooling across the equatorial Pacific in the fast response (fig. S10), rather than the eastern-basin peaked cooling expected from the thermostat mechanism, casting doubt on its role. A recent study proposes that this fast basin-wide cooling is instead driven by intensified trade easterlies resulting from an enhanced land-ocean warming contrast, which is supported by a simulation in which CO_2_ is increased only over land (*36*). If the ocean dynamical thermostat were the primary driver, applying CO_2_ forcing only to the ocean should yield a fast-cooling response; instead, that simulation produces fast warming in the eastern equatorial Pacific, further challenging the thermostat hypothesis. That said, the mechanism underlying the fast response remains unsettled.

Decomposition of the deep tropical response in the 1%-per-year CO_2_ increase simulation shows that the slow component emerges clearly only after around year 80, when it becomes significantly larger than the fast component (Fig. 5 and fig. S12). Comparing the CO_2_ concentration at this transition with present-day values suggests that current conditions remain dominated by the fast response, which is likely to persist for another ∼50 years. This may partially explain why observations show an expansion of the deep tropics in recent decades, in contrast to the long-term projections of contraction (Fig. 1). Coupled historical simulations, by contrast, tend to show contraction, consistent with long-term projections but at odds with observations (Fig. 1), reflecting the well-known model-observation discrepancy in tropical Pacific warming trends (*37*). Our results suggest that this discrepancy may stem from a misrepresentation of the fast response to CO_2_ forcing: The CO_2_-driven fast response may be absent, too weak, or too short-lived in other models. A multimodel intercomparison of large-ensemble abrupt4xCO_2_ experiments is therefore strongly recommended to evaluate this behavior.

One may question whether the historical trends in our model [MPI-ESM-LR; (*26*)], which robustly simulates the CO_2_-driven fast expansion of the deep tropics, better align with observations. Our model is indeed among those that most closely reproduce the observed trends (indicated by the white-contoured historical (HIST) symbol in Fig. 1), but the 50-member ensemble mean of its historical simulation nevertheless shows a slight contraction of 0.2°, with an inter-ensemble standard deviation of 0.4°. This points to the important roles of internal variability and, potentially, aerosol forcing in shaping the historical response. In addition, the model-observation discrepancy may arise from missing Antarctic meltwater (*31*), the absence of Southern Ocean cooling (*38*), and biases in the response to, or representation of, aerosol forcing (*30*).

## MATERIALS AND METHODS

### Reanalysis data

We analyze two reanalysis datasets [European Center for Medium-Range Weather Forecasts Reanalysis v5 (ERA5) (*39*) and National Centers for Environmental Prediction-Department of Energy (NCEP-DOE) (*40*)] for the period between 1979–2024. Recent changes are quantified as the difference between the mean of the last decade (2015–2024) and the first decade (1979–1988).

### CMIP6 data

Historical changes and long-term future projections are illustrated using the HIST and Shared Socioeconomic Pathway 5-8.5 (SSP5-8.5) experiments from 29 coupled global climate models (*41*): ACCESS-CM2, ACCESS-ESM1-5, AWI-CM-1-1-MR, BCC-CSM2-MR, CAMS-CSM1-0, CESM2, CESM2-WACCM, CIESM, CMCC-CM2-SR5, CNRM-CM6-1, CNRM-CM6-1-HR, CNRM-ESM2-1, CanESM5, EC-Earth3-Veg, FGOALS-f3-L, FGOALS-g3, GFDL-CM4, GFDL-ESM4, GISS-E2-1-G, GISS-E2-1-H, INM-CM4-8, IPSL-CM6A-LR, MIROC-ES2L, MIROC6, MPI-ESM1-2-HR, MPI-ESM1-2-LR, MRI-ESM2-0, NorESM2-LM, and NorESM2-MM. For most models, we use the “r1i1p1f1” run, with the exception of CNRM-CM6-1, CNRM-CM6-1-HR, CNRM-ESM2-1, GISS-E2-1-G, and MIROC-ES2L, for which the “r1i1p1f2” run is used. Historical changes are quantified using the same time periods as the reanalysis data, and long-term future changes are defined as the difference between the preindustrial control and the last 20-year mean (2080–2099). In addition, we use the 1%-per-year CO_2_ increase simulation from the “r1i1p1f1” run of MPI-ESM-LR to approximate realistic forcing conditions and to examine how the relative contributions of the fast and slow components evolve over time.

### Abruptly quadrupled CO_2_ simulation

To investigate the timescale-dependent forced responses, we conduct a 30-member ensemble of abrupt4xCO_2_ using MPI-ESM-LR (*26*). The control simulation is integrated for 600 years under preindustrial CO_2_ concentrations with constant preindustrial forcings (solar irradiance, aerosols, land use, etc.). For the quadrupled CO_2_ simulation, each ensemble member branches from the control simulation at 10-year intervals, with CO_2_ concentration instantaneously quadrupled globally from the control state while maintaining all other forcings unchanged. The simulations are then extended for 150 years. Climate responses are calculated as the difference between each ensemble member and its corresponding control period.

### Deep tropical width

Here, deep tropical width is defined as the distance between the tropical meridional centroids of zonal-mean pressure velocity in each hemisphere (fig. S3)

$$\phi_{\text{width}}=\phi_{N}-\phi_{S},\;\text{where}\;\phi_{N/S}=\frac{\int_{0}^{\phi_{0}}\omega_{\text{mid}}^{↑}\phi \;\text{dϕ}}{\int_{0}^{\phi_{0}}\omega_{\text{mid}}^{↑}\;\text{dϕ}}$$

where $\omega_{\text{mid}}^{↑}$ is the upward motion averaged between 300 and 700 hPa, with downward motion set to zero. ϕ is the latitude, and $\phi_{0}=\pm 20{}^{\circ}$ defines the northern and southern tropical boundaries.

### Ocean mixed layer heat budget

We quantify the factors that determine changes in tropical relative SST using the ocean mixed layer heat budget decomposition (*42*, *43*). Neglecting the storage term, the energy budget can be expressed as

$$\begin{array}{c} ∆T_{s}=\frac{1}{\alpha {\bar{Q}}_{\text{lh}}}\cdot \left(∆D_{o}-∆Q_{\text{sw}}-∆Q_{\text{lw}}-∆Q_{\text{sh}}-∆Q_{\text{lh},\text{others}}\right)\\ =∆T_{\text{OHU}}+∆T_{\text{sw}}+∆T_{\text{lw}}+∆T_{\text{sh}}+∆T_{\text{lh},\text{others}} \end{array}$$

where ∆ denotes the difference between the quadrupled CO_2_ and preindustrial control experiments, and the overbar represents the climatology of the preindustrial control. Upward fluxes are defined as positive, indicating atmospheric heating or surface cooling. The term $∆Q_{\mathrm{LH},\text{others}}$ represents the residual between the total latent heat flux change ($∆Q_{\mathrm{LH}}$) and the portion related to Newtonian cooling ($∆Q_{\mathrm{LH},T_{s}}=\alpha {\bar{Q}}_{\mathrm{LH}}\cdot ∆T_{s}$, where $\alpha \equiv \frac{L_{v}}{R_{v}{\bar{T}}_{s}^{2}}\approx 0.06\;K^{-1}$). Hence, the SST change can be attributed to changes in ocean heat uptake $∆T_{\text{OHU}}$, shortwave $∆T_{\mathrm{SW}}$ and longwave $∆T_{\mathrm{LW}}$ radiation, sensible heat flux $∆T_{\mathrm{SH}}$, and latent heat flux driven by changes in wind, relative humidity, and atmospheric stability $∆T_{\mathrm{LH},\text{others}}$.

### Fast and slow decomposition of climate response to 1%-per-year CO_2_ forcing

In the 1%-per-year CO_2_ experiment, the contributions of the fast and slow responses to tropical warming are estimated based on the spatial patterns of tropical SST warming derived from the abrupt quadrupling of CO_2_ experiment (*22*, *23*). For each year, the annual-mean tropical SST warming pattern is regressed onto the normalized fast and slow SST responses (fig. S11, A and B). The fast SST response is defined as average response over years 1–10, while the slow SST response is defined as the difference between the long-term response (years 111–150) and the fast response. This multiple linear regression can be expressed as

$$∆T_{\text{pct}}\left(t,x,y\right)=F\left(t\right)\cdot \frac{∆T_{\text{fast}}\left(x,y\right)}{∆{\bar{T}}_{\text{fast}}}+S\left(t\right)\cdot \frac{∆T_{\text{slow}}\left(x,y\right)}{∆{\bar{T}}_{\text{slow}}}+R\left(t\right)$$

where $∆T_{\text{pct}}$ is the annual-mean tropical SST change in 1%-per-year CO_2_ experiment, and $∆T_{\text{fast}}$ and $∆T_{\text{slow}}$ are the fast and slow SST warming patterns (fig. S11, A and B). Here, ∆ denotes the change relative to the preindustrial control, and the overbars indicate the tropical mean (20°S to 20°N). The regression coefficients, F(t) and S(t), represent the time-varying contributions of the fast and slow components, respectively, while the residual term R(t) is linearly independent of the two components. The physical meanings of F(t), S(t), and R(t) are their respective contributions to the tropical-mean warming, with units in kelvin.

The coefficients F(t) and S(t) can be further used to reconstruct the fast and slow components of other variables in the 1%-per-year CO_2_ experiment, such as the equatorial vertical motion $\omega_{\text{eq}}$ or the deep tropical width $\phi_{\text{width}}$. The anomaly of a reconstructed variable Y can be written as

$$∆Y_{\text{pct}}\left(t,x,y\right)=F\left(t\right)\cdot \frac{∆Y_{\text{fast}}\left(x,y\right)}{∆{\bar{T}}_{\text{fast}}}+S\left(t\right)\cdot \frac{∆Y_{\text{slow}}\left(x,y\right)}{∆{\bar{T}}_{\text{slow}}}$$

where $∆Y_{\text{fast}}$ and $∆Y_{\text{slow}}$ in the numerators are the fast and slow responses of either $∆{\text{SST}}_{\text{relt}}$ or $∆\omega_{\text{mid}}$, as shown in fig. S11. The right two terms in the above equation represent the fast and slow components, expressed as the fast and slow responses normalized by the tropical-mean SST change and multiplied by their respective time-varying regression coefficients. For $∆\phi_{\text{width}}$, however, additional steps are required. Since the deep tropical width is diagnosed from absolute fields, rather than anomalies, the fast and slow components of anomalous pressure velocity are added back to the piControl state to reconstruct the corresponding absolute fields. These reconstructed fields are then used to compute the deep tropical width, from which anomalies ($∆\phi_{\text{width}}$ relative to piControl) are subsequently derived.

## Supplementary Materials

### This PDF file includes:

Figs. S1 to S12 References
