# Supplementary material for: Fast expansion and slow contraction of the ITCZ in response to CO2 forcing
Source: Sci Adv. 2026 Jul 23;12(30):eaef2833. doi: 10.1126/sciadv.aef2833 (PMC13394389; doi:10.1126/sciadv.aef2833)
Supplement: Supplementary file 1 [file sciadv.aef2833_sm.pdf]

Science Advances
AAAS

Supplementary Materials for

Fast expansion and slow contraction of the ITCZ in response to CO_2 forcing

Jiayu Zhang and Sarah M. Kang

Corresponding author: Jiayu Zhang, jiayu.zhang@mpimet.mpg.de

Sci. Adv. **12**, eaef2833 (2026)
DOI: 10.1126/sciadv.aef2833

This PDF file includes:

Figs. S1 to S12
References

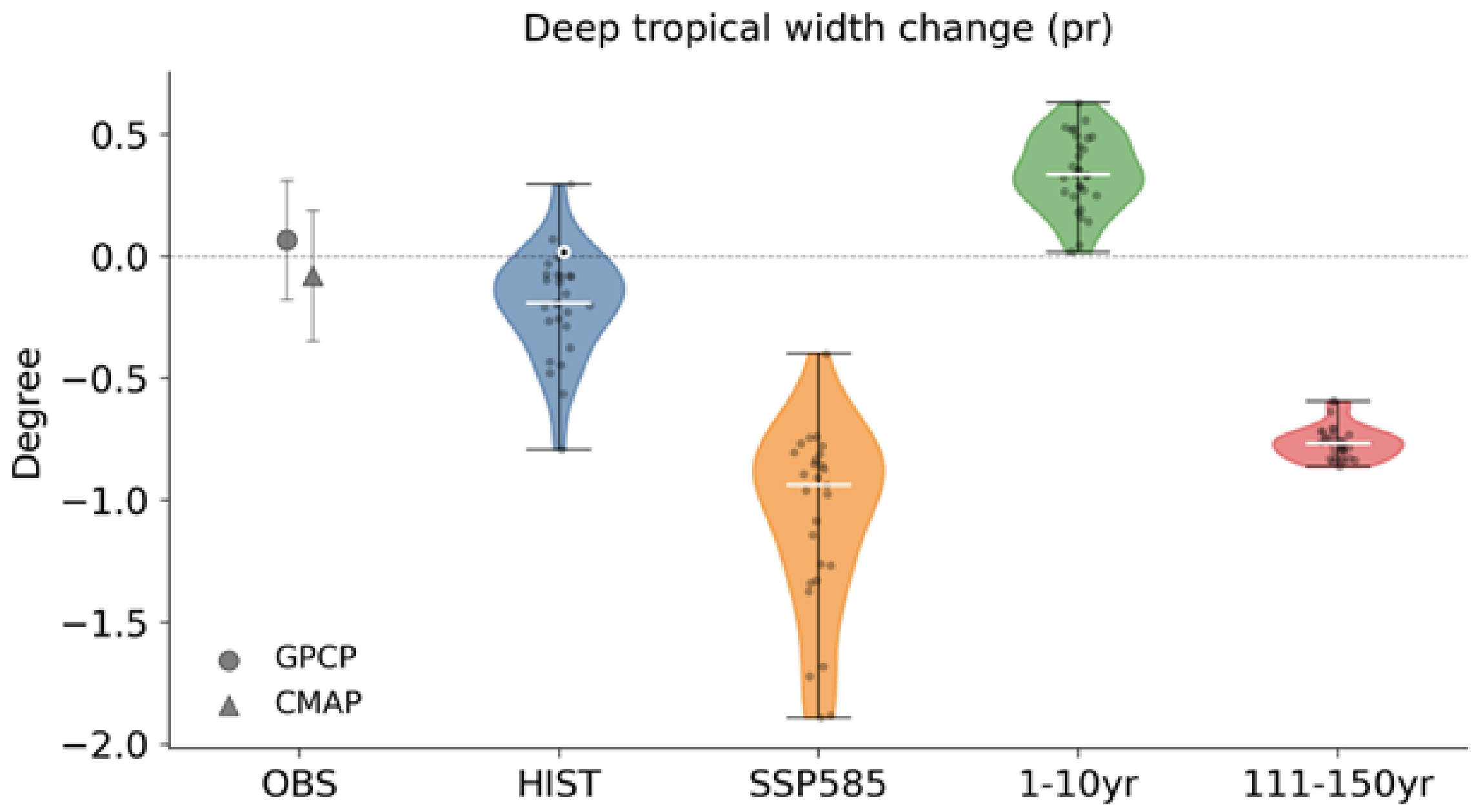

Fig. S1. Deep-tropical width changes in different scenarios. Same as Fig. 1, but for the deep tropical width defined using precipitation (refer to Fig. S5A). Two observational precipitation datasets (GPCP *(44)* and CMAP *(45)*) are used.

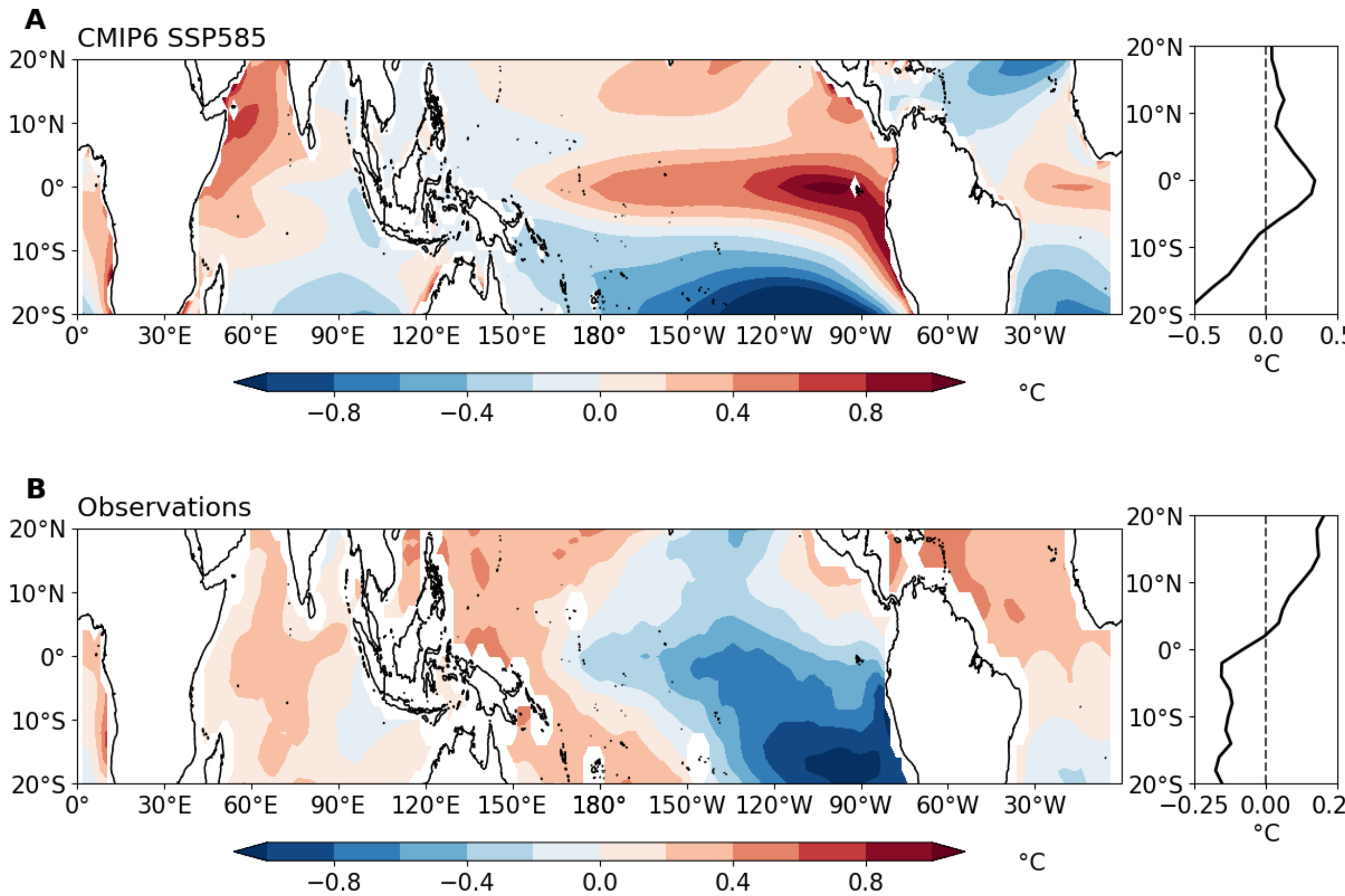

Fig. S2. Tropical relative SST changes at the end of this century and in the recent decades. **A,** Spatial distribution of the SST anomalies, with the 20°S-20°N mean removed, in CMIP6 at the end of century under SSP5-8.5 scenario, calculated as the difference between the years 2080–2099 and the last 200 years of the pre-industrial, with the corresponding zonal mean shown on the right. **B,** As in **A,** but showing the linear SST trends over 1979–2024, averaged across four datasets (HadISST (*46*), COBE2 (*47*), ERSST.V5 (*48*) and ORAS5 (*49*)) and expressed as 45-year changes (unit: °C).

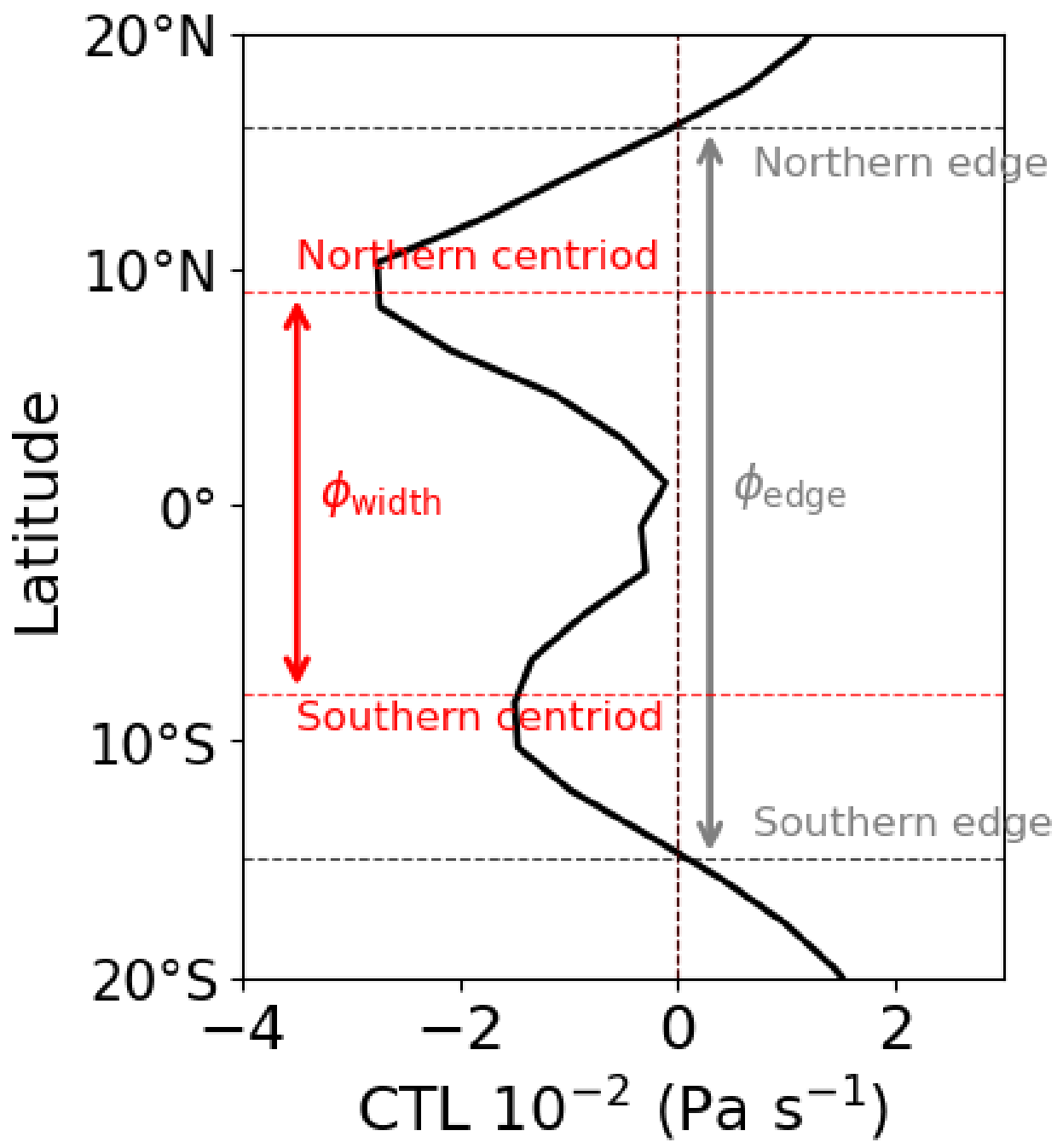

Fig. S3. Schematic diagram of deep tropical width. The black solid line is the zonal-mean pressure velocity averaged between 300–700 hPa (ω_{mid}) in the control simulation. The two horizontal red dashed lines mark the northern and southern centroids, with their separation defining the deep tropical width (ϕ_{width}). The two horizontal gray dashed lines mark the northern and southern edges of upward motion ($\omega_{\mathrm{mid}} = 0$), with their latitudinal difference defining the tropical edge (ϕ_{edge}).

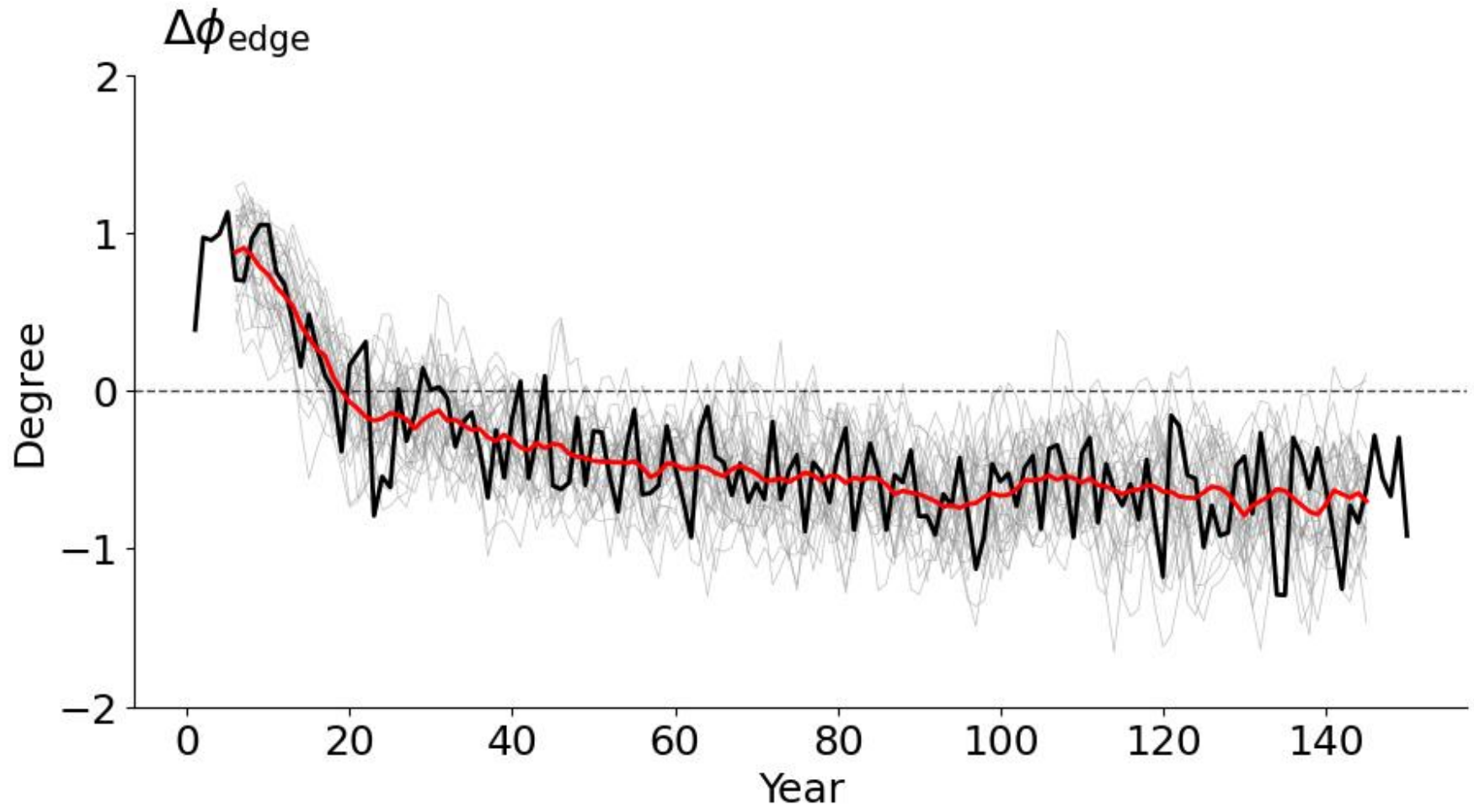

Fig. S4. Evolution of tropical edge change to abruptly quadrupled CO_2. Similar to Fig. 2A, but for the tropical edge (ϕ_{edge}, refer to Fig. S3); positive values indicate poleward expansion and negative values contraction.

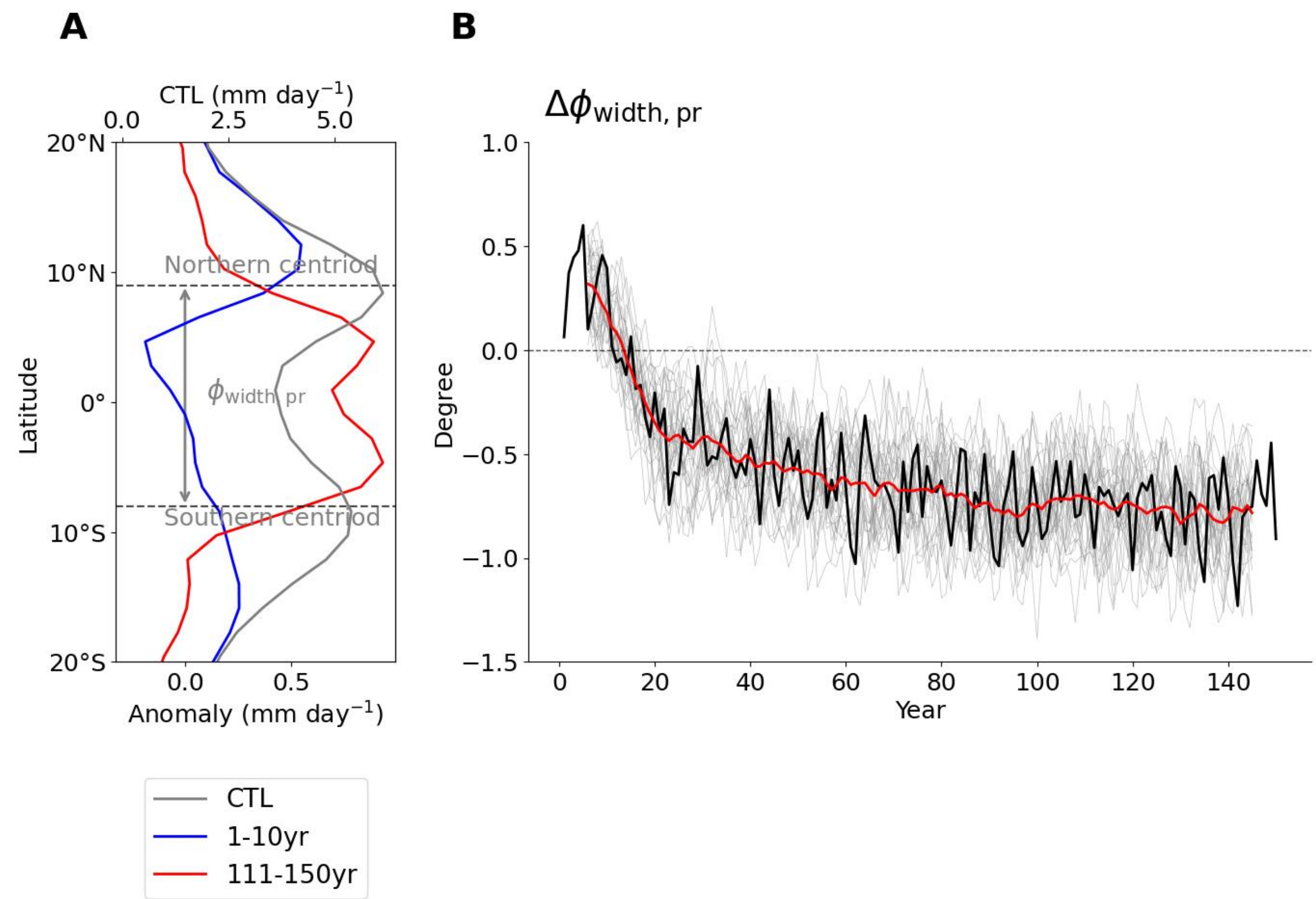

Fig. S5. Evolution of zonal-mean tropical precipitation to abruptly quadrupled CO_2. A, Zonal-mean precipitation in the control simulation (grey) and its response in the fast (years 1-10; blue) and long-term (years 111-150; red) periods. The separation of northern and southern centroids of zonal-mean precipitation defines $\phi_{\text{width,pr}}$. **B,** Similar to Fig. 2A, but for the tropical width defined by precipitation ($\phi_{\text{width,pr}}$; positive values indicate poleward expansion and negative values contraction).

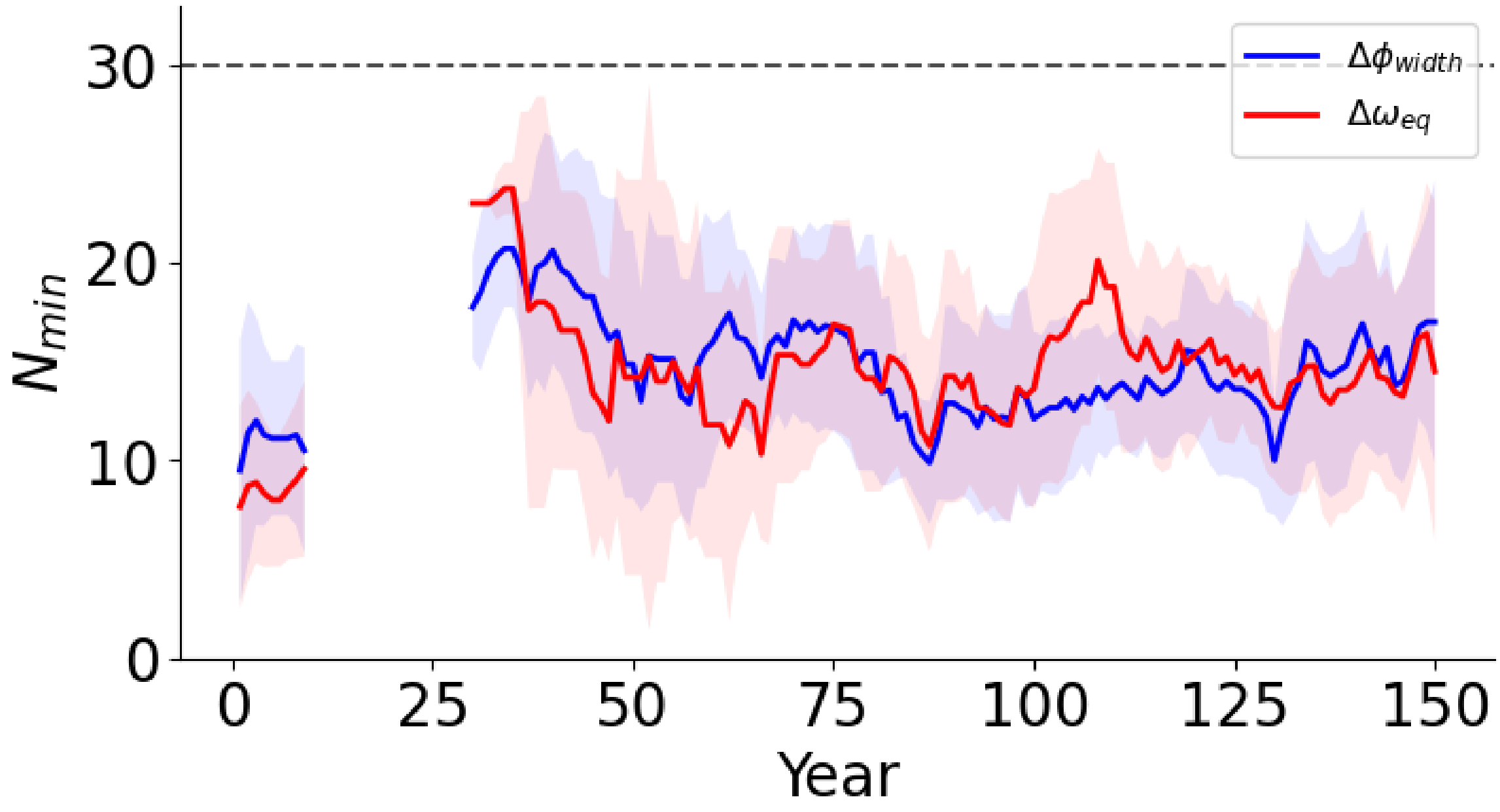

Fig. S6. Time series of minimum ensemble size (N_{min}) required to detect the forced response. Timeseries of N_{min} for the deep tropical width (blue) and equatorial pressure velocity amplitude (red). N_{min} is estimated from the standard normal (Z) distribution as $N_{\mathrm{min}}(t) = \left[\frac{z_{\alpha/2}\cdot\sigma(t)}{|S(t)|}\right]^2$, where $S(t)$ is the 30-member ensemble mean, $\sigma(t)$ the inter-ensemble standard deviation, and the significant level is set to $\alpha = 0.05$. The solid line shows the 11-year running mean of raw values, and shading indicates the 95% confidence interval based on variability within each 11-year window. Years 15 to 25 are masked because the forced responses are relatively small during this transition phase.

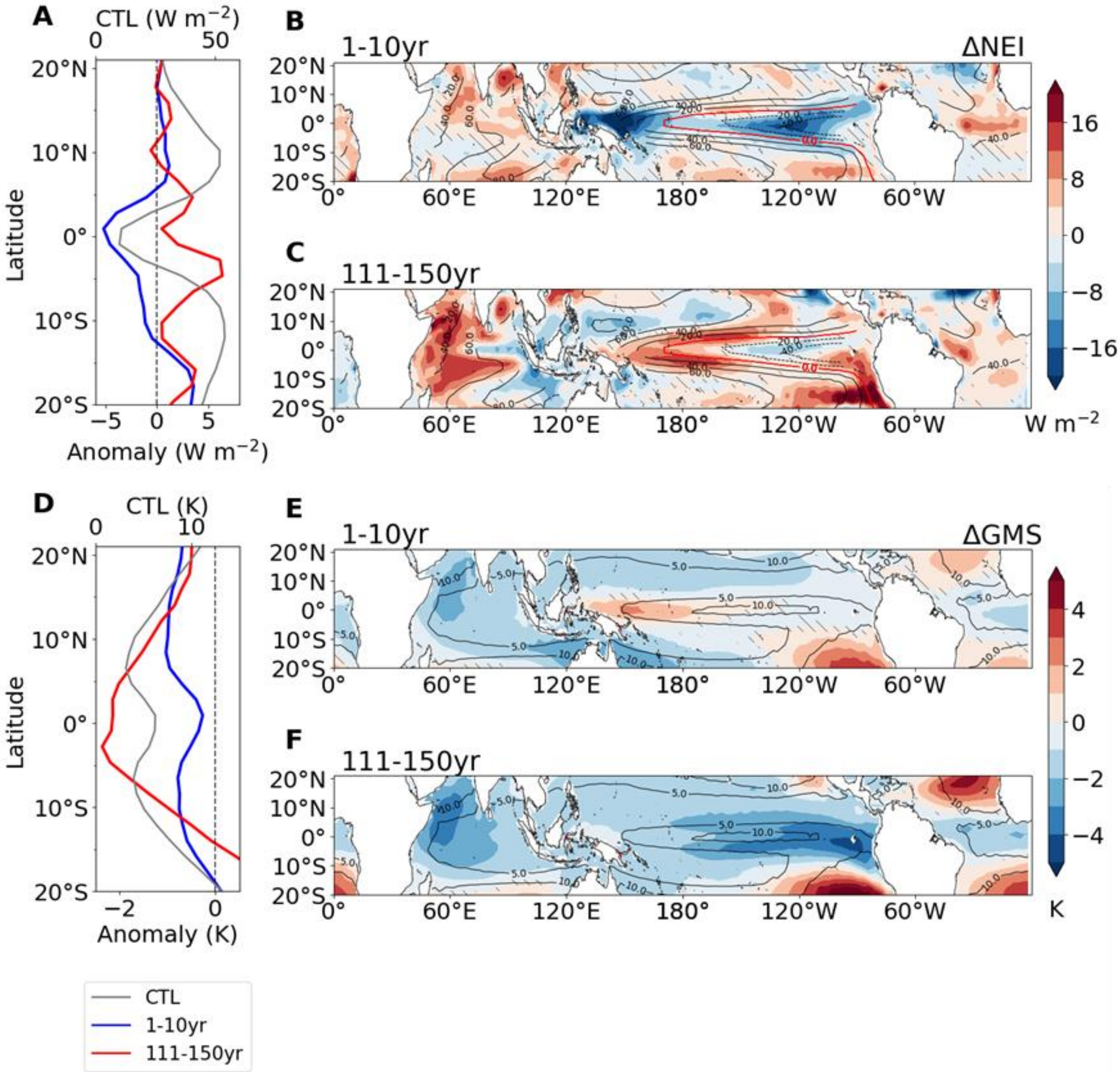

Fig. S7. Fast and long-term responses of atmospheric net energy input (NEI) and gross moist stability (GMS). A, Zonal-mean NEI in the pre-industrial climatology (gray) and anomalies in the fast (years 1-10; blue) and long-term (years 111-150; red) periods. NEI is measured as the difference between the net energy fluxes at the top and bottom of the atmosphere. **B** and **C**, Spatial patterns of NEI anomalies (shading) in the fast and long-term periods, respectively, and the pre-industrial climatology (contours). **D-F**, Same as **A-C**, but for GMS, computed as the difference between the moist static energy (MSE $= C_pT + gz + L_vq$) in the upper (150-300hPa) and lower (850-925hPa) tropospheric averages, in the unit of temperature after normalizing by C_p. Hatched regions indicate where values are not significant at the 95% confidence level according to the t-test.

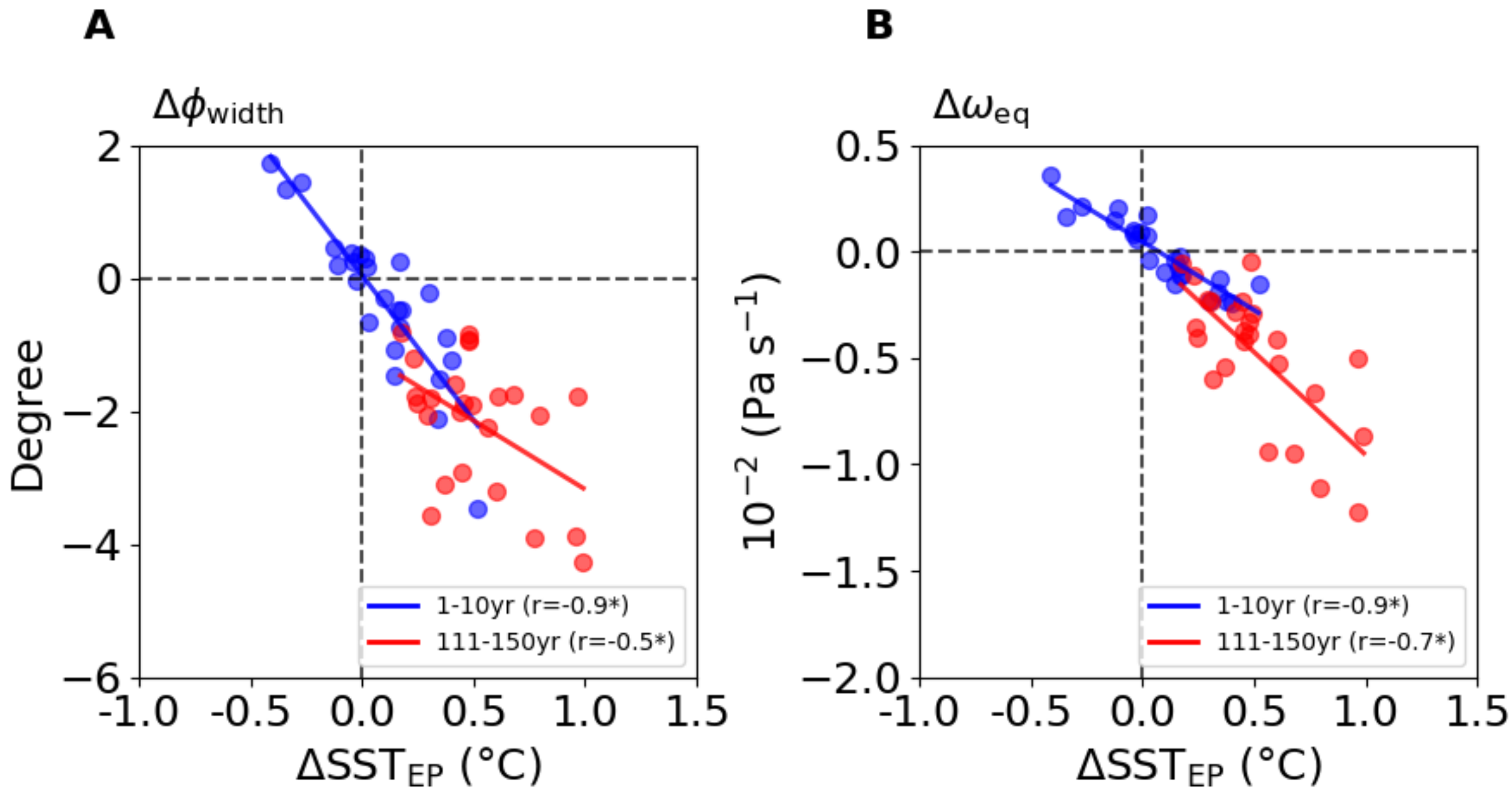

Fig. S8. Inter-model correlations between tropical SST warming patterns and deep tropical changes in CMIP6 models under abruptly quadrupled CO_2 forcing. Anomalies in **A,** deep tropical width (ϕ_{width}; positive values indicate poleward expansion and negative values equatorward contraction) and **B,** equatorial pressure velocity averaged between 300–700 hPa (ω_{eq}; positive values indicate weakening and negative values strengthening), plotted against ΔSST_{EP} (relative SST anomaly in the box shown in Fig. 3B). Blue and red circles represent the fast (years 1-10) and long-term (years 111-150) responses, respectively. Solid lines show the intermodel regressions for each period, with correlation coefficients given in the bottom-right corner. Asterisks denote correlations significant at the 95% confidence level.

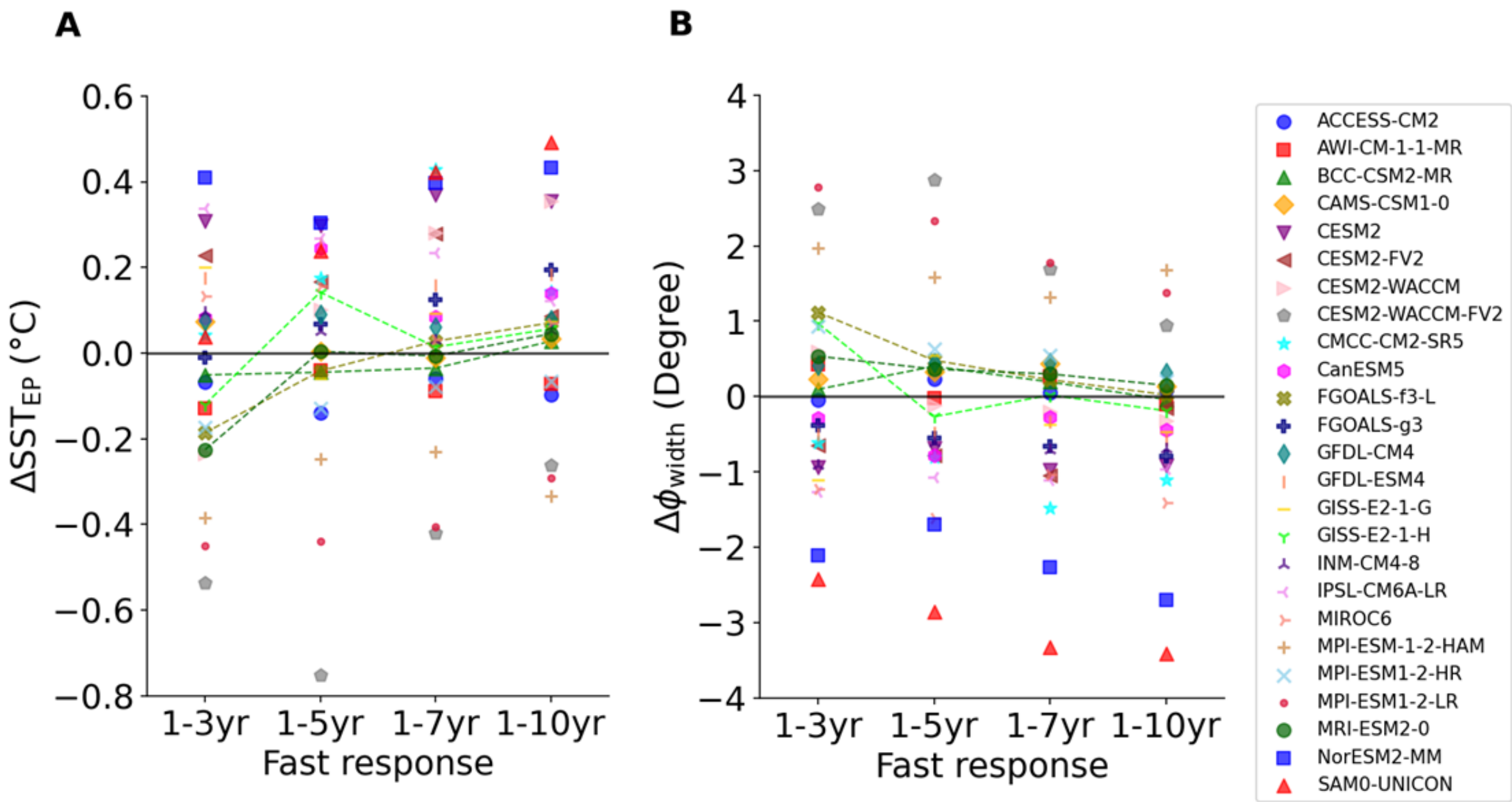

Fig. S9. Fast responses estimated using different averaging windows in CMIP6 models under abruptly quadrupled CO_2 forcing. A, Relative SST change in the equatorial Pacific (ΔSST_{EP}; relative SST anomaly within the box shown in Fig. 3B), and **B,** deep tropical width change ($\Delta\phi_{width}$; positive values indicate poleward expansion and negative values equatorward contraction). In both panels, the x-axis denotes the different averaging windows (e.g., the first 3, 5, 7, or 10 years). Each dot represents an individual CMIP6 model, with model names listed on the right. Dashed lines highlight four example models (BCC-CSM2-MR, FGOALS-f3-L, GISS-E2-1-H and MRI-ESM2-0), for which the sign of the fast response depends on the chosen time window.

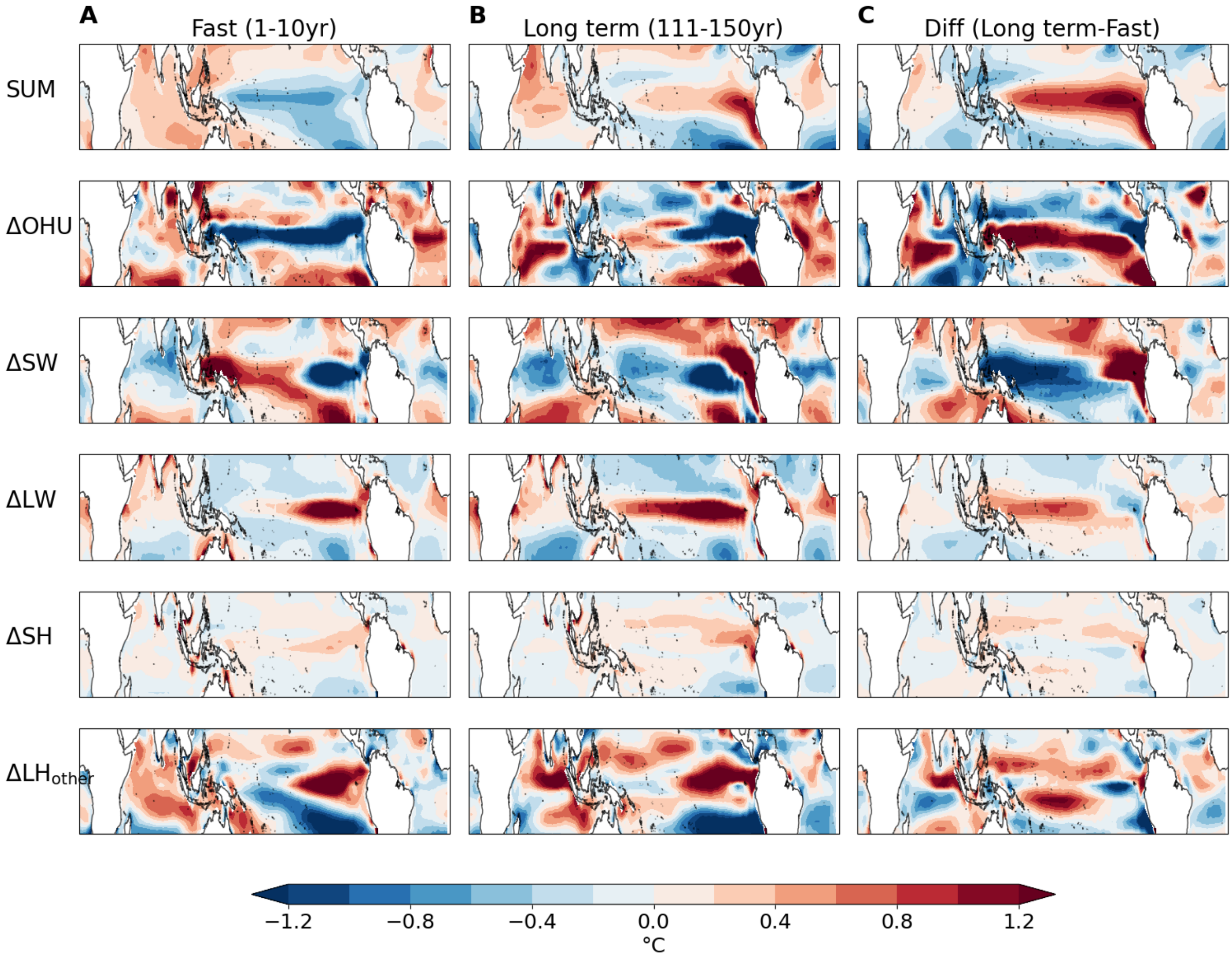

Fig. S10. Spatial patterns of each term in ocean mixed layer heat budget over the tropics. **A,** Fast response (1-10yr), **B,** long-term response (111-150yr), and **C,** the difference between the long-term and fast responses.

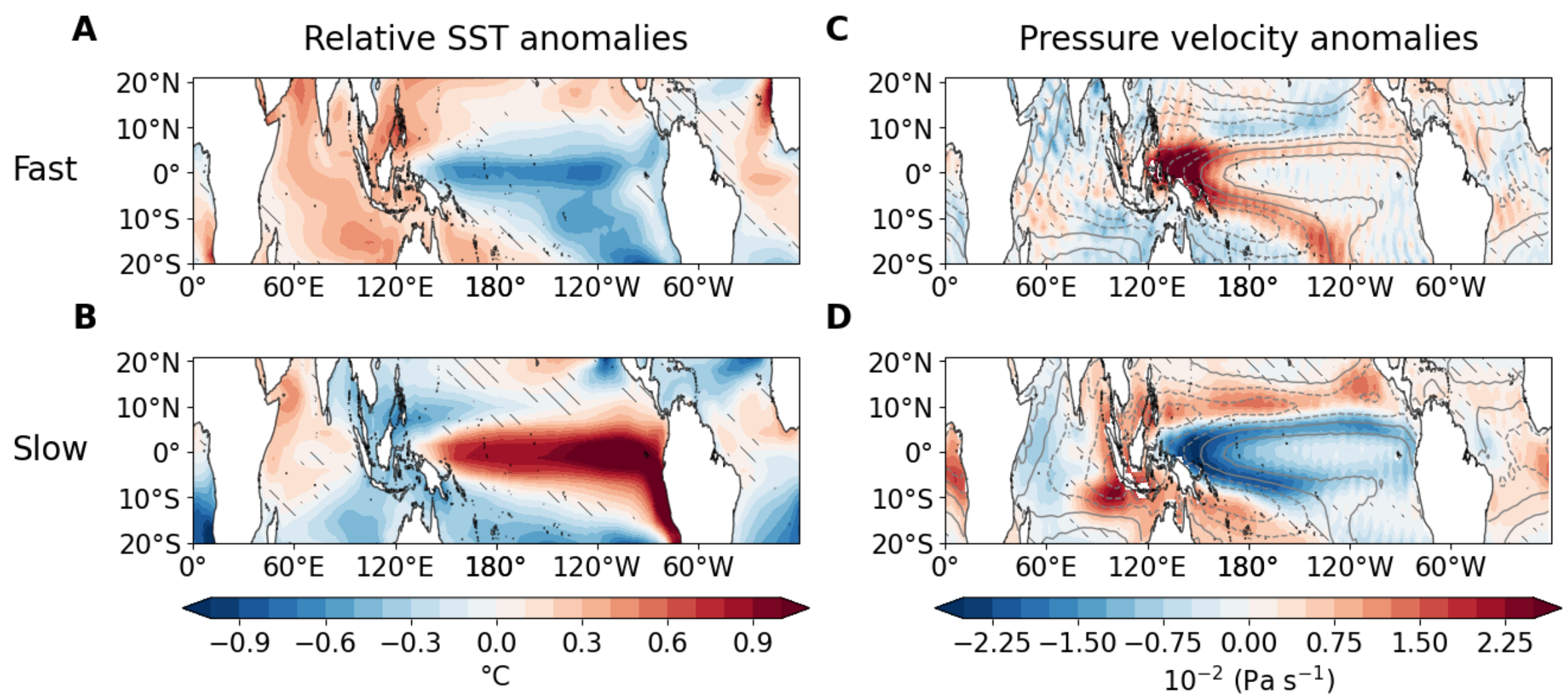

Fig. S11. Fast and slow responses defined according to abrupt4xCO2 simulation. A and **B,** Relative SST anomalies (ΔSST_{relt}), **C** and **D,** pressure velocity anomalies averaged between 300–700 hPa in shading ($\Delta\omega_{mid}$, positive: downward, negative: upward) and climatological mean pressure velocity in contours (dashed: upward, solid: downward). Hatched regions indicate where values are not significant at the 95% confidence level according to the t-test.

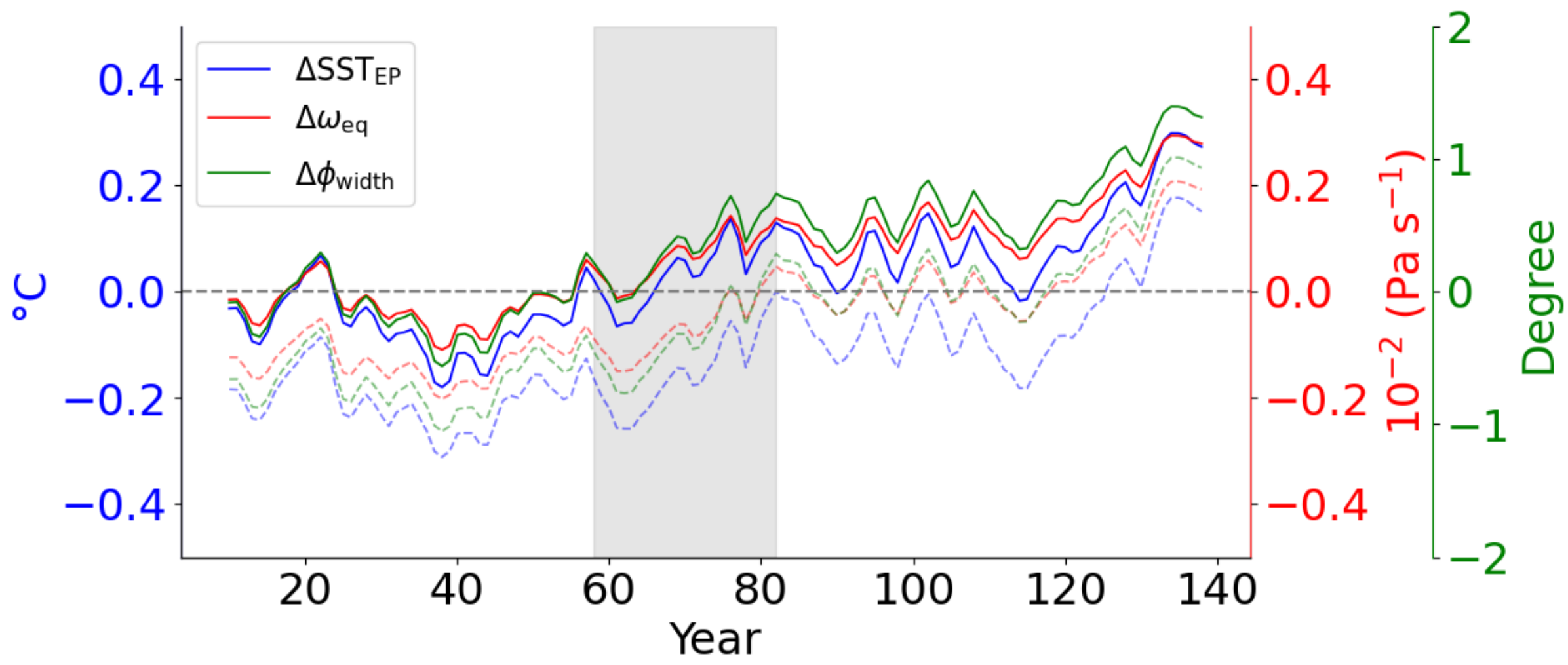

Fig. S12. Relative contribution of the fast and slow components in 1%-per-year CO_2 simulation. The solid line represents the difference between the fast and slow components of each variable (blue and red lines in Fig. 5). The dashed line shows the same difference accounting for uncertainty, calculated as the difference between the lower bound of the slow (fast) component and the upper bound of the fast (slow) component. Negative values indicate dominance of the fast component; positive values indicate dominance of the slow component. The transition occurs between years 60 and 80, as indicated by the gray shading, when the lines cross zero.

REFERENCES

1. O. Watt-Meyer, D. M. W. Frierson, ITCZ width controls on hadley cell extent and eddy-driven jet position and their response to warming. *J. Clim.* **32**, 1151–1166 (2019).

2. A. G. Pendergrass, M. P. Byrne, O. Watt-Meyer, P. Maher, M. J. Webb, Impact of ITCZ width on global climate: ITCZ-MIP. *Geosci. Model Dev.* **17**, 6365–6378 (2024).

3. H. Su, J. H. Jiang, J. D. Neelin, T. J. Shen, C. Zhai, Q. Yue, Z. Wang, L. Huang, Y. S. Choi, G. L. Stephens, Y. L. Yung, Tightening of tropical ascent and high clouds key to precipitation change in a warmer climate. *Nat. Commun.* **8**, 15771 (2017).

4. B. Tian, Spread of model climate sensitivity linked to double-Intertropical Convergence Zone bias. *Geophys. Res. Lett.* **42**, 4133–4141 (2015).

5. M. J. Webb, A. P. Lock, Testing a physical hypothesis for the relationship between climate sensitivity and double-ITCZ bias in climate models. *J. Adv. Model. Earth Syst.* **12**, e2019MS001999 (2020).

6. W. K. M. Lau, K.-M. Kim, Robust hadley circulation changes and increasing global dryness due to CO_2 warming from CMIP5 model projections. *Proc. Natl. Acad. Sci. U.S.A.* **112**, 3630–3635 (2015).

7. M. P. Byrne, T. Schneider, Narrowing of the ITCZ in a warming climate: Physical mechanisms. *Geophys. Res. Lett.* **43**, 11350–311357 (2016).

8. M. P. Byrne, T. Schneider, Energetic constraints on the width of the intertropical convergence zone. *J. Clim.* **29**, 4709–4721 (2016).

9. M. P. Byrne, A. G. Pendergrass, A. D. Rapp, K. R. Wodzicki, Response of the intertropical convergence zone to climate change: Location, width, and strength. *Curr. Clim. Change Rep.* **4**, 355–370 (2018).

10. M. P. Byrne, R. Thomas, Dynamics of ITCZ width: Ekman processes, non-Ekman processes, and links to sea surface temperature. *J. Atmos. Sci.* **76**, 2869–2884 (2019).

11. W. Zhou, S.-P. Xie, D. Yang, Enhanced equatorial warming causes deep-tropical contraction and subtropical monsoon shift. *Nat. Clim. Change* **9**, 834–839 (2019).

12. S.-P. Xie, C. Deser, G. A. Vecchi, J. Ma, H. Teng, A. T. Wittenberg, Global warming pattern formation: Sea surface temperature and rainfall. *J. Clim.* **23**, 966–986 (2010).

13. N. Albern, A. Voigt, S. A. Buehler, V. Grützun, Robust and nonrobust impacts of atmospheric cloud-radiative interactions on the tropical circulation and its response to surface warming. *Geophys. Res. Lett.* **45**, 8577–8585 (2018).

14. A. Donohoe, A. R. Atwood, M. P. Byrne, Controls on the width of tropical precipitation and its contraction under global warming. *Geophys. Res. Lett.* **46**, 9958–9967 (2019).

15. M. Popp, N. J. Lutsko, Quantifying the zonal-mean structure of tropical precipitation. *Geophys. Res. Lett.* **44**, 9470–9478 (2017).

16. M. Popp, S. Bony, Stronger zonal convective clustering associated with a wider tropical rain belt. *Nat. Commun.* **10**, 4261 (2019).

17. W. Zhou, L. R. Leung, J. Lu, D. Yang, F. Song, Contrasting recent and future ITCZ changes from distinct tropical warming patterns. *Geophys. Res. Lett.* **47**, e2020GL089846 (2020).

18. A. C. Clement, R. Seager, M. A. Cane, S. E. Zebiak, An ocean dynamical thermostat. *J. Clim.* **9**, 2190–2196 (1996).

19. U. K. Heede, A. V. Fedorov, Eastern equatorial Pacific warming delayed by aerosols and thermostat response to CO_2 increase. *Nat. Clim. Change* **11**, 696–703 (2021).

20. M. Watanabe, S. M. Kang, M. Collins, Y.-T. Hwang, S. McGregor, M. F. Stuecker, Possible shift in controls of the tropical Pacific surface warming pattern. *Nature* **630**, 315–324 (2024).

21. I. M. Held, M. Winton, K. Takahashi, T. Delworth, F. Zeng, G. K. Vallis, Probing the fast and slow components of global warming by returning abruptly to preindustrial forcing. *J. Clim.* **23**, 2418–2427 (2010).

22. P. Ceppi, G. Zappa, T. G. Shepherd, J. M. Gregory, Fast and slow components of the extratropical atmospheric circulation response to CO_2 forcing. *J. Clim.* **31**, 1091–1105 (2018).

23. G. Zappa, P. Ceppi, T. G. Shepherd, Time-evolving sea-surface warming patterns modulate the climate change response of subtropical precipitation over land. *Proc. Natl. Acad. Sci. U.S.A.* **117**, 4539–4545 (2020).

24. U. K. Heede, A. V. Fedorov, N. J. Burls, Time scales and mechanisms for the tropical pacific response to global warming: A tug of war between the ocean thermostat and weaker walker. *J. Clim.* **33**, 6101–6118 (2020).

25. T. Bischoff, T. Schneider, The equatorial energy balance, ITCZ position, and double-ITCZ bifurcations. *J. Clim.* **29**, 2997–3013 (2016).

26. T. Mauritsen, J. Bader, T. Becker, J. Behrens, M. Bittner, R. Brokopf, V. Brovkin, M. Claussen, T. Crueger, M. Esch, I. Fast, S. Fiedler, D. Fläschner, V. Gayler, M. Giorgetta, D. S. Goll, H. Haak, S. Hagemann, C. Hedemann, C. Hohenegger, T. Ilyina, T. Jahns, D. Jimenéz-de-la-Cuesta, J. Jungclaus, T. Kleinen, S. Kloster, D. Kracher, S. Kinne, D. Kleberg, G. Lasslop, L. Kornblueh, J. Marotzke, D. Matei, K. Meraner, U. Mikolajewicz, K. Modali, B. Möbis, W. A. Müller, J. E. M. S. Nabel, C. C. W. Nam, D. Notz, S.-S. Nyawira, H. Paulsen, K. Peters, R. Pincus, H. Pohlmann, J. Pongratz, M. Popp, T. J. Raddatz, S. Rast, R. Redler, C. H. Reick, T. Rohrschneider, V. Schemann, H. Schmidt, R. Schnur, U. Schulzweida, K. D. Six, L. Stein, I. Stemmler, B. Stevens, J.-S. von Storch, F. Tian, A. Voigt, P. Vrese, K.-H. Wieners, S. Wilkenskjeld, A. Winkler, E. Roeckner, Developments in the MPI-M Earth system model version 1.2 (MPI-ESM1.2) and its response to increasing CO_2. *J. Adv. Model. Earth Syst.* **11**, 998–1038 (2019).

27. W. Liu, S. Li, C. Li, M. Rugenstein, A. P. Thomas, Contrasting fast and slow intertropical convergence zone migrations linked to delayed Southern Ocean warming. *Nat. Clim. Change* **14**, 732–739 (2024).

28. P. Huang, S.-P. Xie, K. Hu, G. Huang, R. Huang, Patterns of the seasonal response of tropical rainfall to global warming. *Nat. Geosci.* **6**, 357–361 (2013).

29. J. D. Neelin, I. M. Held, Modeling tropical convergence based on the moist static energy budget. *Mon. Weather Rev.* **115**, 3–12 (1987).

30. Y. T. Hwang, S. P. Xie, P. J. Chen, H. Y. Tseng, C. Deser, Contribution of anthropogenic aerosols to persistent La Niña-like conditions in the early 21st century. *Proc. Natl. Acad. Sci. U.S.A.* **121**, e2315124121 (2024).

31. Y. Dong, A. G. Pauling, S. Sadai, K. C. Armour, Antarctic ice-sheet meltwater reduces transient warming and climate sensitivity through the sea-surface temperature pattern effect. *Geophys. Res. Lett.* **49**, e2022GL101249 (2022).

32. Y. Dong, L. M. Polvani, Y.-T. Hwang, M. R. England, Stratospheric ozone depletion has contributed to the recent tropical La Niña-like cooling pattern. *NPJ Clim. Atmos. Sci.* **8**, 150 (2025).

33. M. H. Bordbar, T. Martin, M. Latif, W. Park, Role of internal variability in recent decadal to multidecadal tropical Pacific climate changes. *Geophys. Res. Lett.* **44**, 4246–4255 (2017).

34. U. K. Heede, A. V. Fedorov, N. J. Burls, A stronger versus weaker Walker: Understanding model differences in fast and slow tropical Pacific responses to global warming. *Clim. Dyn.* **57**, 2505–2522 (2021).

35. K. Lu, J. He, B. Fosu, M. Rugenstein, Mechanisms of fast walker circulation responses to CO_2 forcing. *Geophys. Res. Lett.* **48**, e2021GL095708 (2021).

36. M. Günther, S. M. Kang, Y. Kaspi, Heating the land cools the eastern and equatorial Pacific. *Sci. Adv.* **12**, eaeb7004 (2026).

37. R. C. J. Wills, Y. Dong, C. Proistosecu, K. C. Armour, D. S. Battisti, Systematic climate model biases in the large-scale patterns of recent sea-surface temperature and sea-level pressure change. *Geophys. Res. Lett.* **49**, e2022GL100011 (2022).

38. S. M. Kang, P. Ceppi, Y. Yu, I.-S. Kang, Recent global climate feedback controlled by Southern Ocean cooling. *Nat. Geosci.* **16**, 775–780 (2023).

39. H. Hersbach, B. Bell, P. Berrisford, S. Hirahara, A. Horányi, J. Muñoz-Sabater, J. Nicolas, C. Peubey, R. Radu, D. Schepers, A. Simmons, C. Soci, S. Abdalla, X. Abellan, G. Balsamo, P. Bechtold, G. Biavati, J. Bidlot, M. Bonavita, G. De Chiara, P. Dahlgren, D. Dee, M. Diamantakis, R. Dragani, J. Flemming, R. Forbes, M. Fuentes, A. Geer, L. Haimberger, S. Healy, R. J. Hogan, E. Hólm, M. Janisková, S. Keeley, P. Laloyaux, P. Lopez, C. Lupu, G. Radnoti, P. de Rosnay, I. Rozum, F. Vamborg, S. Villaume, J.-N. Thépaut, The ERA5 global reanalysis. *Q. J. Roy. Meteorol. Soc.* **146**, 1999–2049 (2020).

40. M. Kanamitsu, W. Ebisuzaki, J. Woollen, S.-K. Yang, J. J. Hnilo, M. Fiorino, G. L. Potter, NCEP–DOE AMIP-II reanalysis (R-2). *Bull. Am. Meteorol. Soc.* **83**, 1631–1644 (2002).

41. V. Eyring, S. Bony, G. A. Meehl, C. A. Senior, B. Stevens, R. J. Stouffer, K. E. Taylor, Overview of the coupled model intercomparison project phase 6 (CMIP6) experimental design and organization. *Geosci. Model Dev.* **9**, 1937–1958 (2016).

42. S. M. Kang, S.-P. Xie, Y. Shin, H. Kim, Y.-T. Hwang, M. F. Stuecker, B. Xiang, M. Hawcroft, Walker circulation response to extratropical radiative forcing. *Sci. Adv.* **6**, eabd3021 (2020).

43. Y.-T. Hwang, S.-P. Xie, C. Deser, S. M. Kang, Connecting tropical climate change with Southern Ocean heat uptake. *Geophys. Res. Lett.* **44**, 9449–9457 (2017).

44. R. F. Adler, G. J. Huffman, A. Chang, R. Ferraro, P. Xie, J. Janowiak, B. Rudolf, U. Schneider, S. Curtis, D. Bolvin, A. Gruber, J. Susskind, P. Arkin, The version 2 global precipitation climatology project (GPCP) monthly precipitation analysis (1979-present). *J. Hydrometeorol.* **4**, 1147–1167 (2003).

45. P. Xie, P. A. Arkin, Global precipitation: A 17-year monthly analysis based on gauge observations, satellite estimates, and numerical model outputs. *Bull. Am. Meteor. Soc.* **78**, 2539–2558 (1997).

46. N. A. Rayner, D. E. Parker, E. B. Horton, C. K. Folland, L. V. Alexander, D. P. Rowell, E. C. Kent, A. Kaplan, Global analyses of sea surface temperature, sea ice, and night marine air temperature since the late nineteenth century. *J. Geopys. Res. Atmos.* **108**, 4407 (2003).

47. S. Hirahara, M. Ishii, Y. Fukuda, Centennial-scale sea surface temperature analysis and its uncertainty. *J. Clim.* **27**, 57–75 (2014).

48. B. Y. Huang, P. W. Thorne, V. F. Banzon, T. Boyer, G. Chepurin, J. H. Lawrimore, M. J. Menne, T. M. Smith, R. S. Vose, H. M. Zhang, Extended reconstructed sea surface temperature, version 5 (ERSSTv5): Upgrades, validations, and intercomparisons. *J. Clim.* **30**, 8179–8205 (2017).

49. H. Zuo, M. A. Balmaseda, S. Tietsche, K. Mogensen, M. Mayer, The ECMWF operational ensemble reanalysis–analysis system for ocean and sea ice: A description of the system and assessment. *Ocean Sci.* **15**, 779–808 (2019).
